# Ionizable liposomal siRNA therapeutics enables potent and persistent treatment of Hepatitis B

**DOI:** 10.1038/s41392-021-00859-y

**Published:** 2022-02-11

**Authors:** Yuanyu Huang, Shuquan Zheng, Zhaoxu Guo, Xavier de Mollerat du Jeu, Xing-Jie Liang, Zhiwei Yang, Hong-Yan Zhang, Shan Gao, Zicai Liang

**Affiliations:** 1 Suzhou Ribo Life Science Co. Ltd., Jiangsu, 215300 China; 2 Advanced Research Institute of Multidisciplinary Science, School of Life Science, School of Medical Technology (Institute of Engineering Medicine), Key Laboratory of Molecular Medicine and Biotherapy, Key Laboratory of Medical Molecule Science and Pharmaceutics Engineering, Beijing Institute of Technology, Beijing, 100081 China; 3 Thermo Fisher Scientific, Carlsbad, CA USA; 4 Center for Excellence in Nanoscience and CAS Key Laboratory for Biomedical Effects of Nanomaterials and Nanosafety, Chinese Academy of Sciences (CAS), National Center for Nanoscience and Technology, Beijing, 100190 China

**Keywords:** Nanobiotechnology, Drug delivery, Nucleic-acid therapeutics, Oligo delivery, Infectious diseases

## Abstract

Small interfering RNA (siRNA) constitutes a promising therapeutic modality supporting the potential functional cure of hepatitis B. A novel ionizable lipidoid nanoparticle (RBP131) and a state-of-the-art lyophilization technology were developed in this study, enabling to deliver siRNA targeting apolipoprotein B (APOB) into the hepatocytes with an ED_50_ of 0.05 mg/kg after intravenous injection. In addition, according to the requirements of Investigational New Drug (IND) application, a potent siRNA targeting hepatitis B virus (HBV) was selected and encapsulated with RBP131 to fabricate a therapeutic formulation termed RB-HBV008. Efficacy investigations in transient and transgenic mouse models revealed that the expressions of viral RNAs and antigens (HBsAg and HBeAg), as well as viral DNA, were repressed, dose-dependently and time-dependently at multilog decreasing amplitude, in both circulation and liver tissue. In contrast, entecavir (ETV), the first-line clinically-employed nucleoside analog drug, barely recused the antigen expression, although it triggered as high as 3.50 log reduction of viral DNA, in line with clinical observations. Moreover, the toxicity profiles suggested satisfactory safety outcomes with ten times the therapeutic window. Therefore, this study provides an effective nucleic acid delivery system and a promising RNAi agent for the treatment of hepatitis B.

## Introduction

Hepatitis B caused by the hepatitis B virus (HBV) that affects the liver is a global public health threat that causes considerable liver-related morbidity and mortality. About a third of the world’s population has been infected at one point in their lives, including 343 million who have chronic infections. There is annual 600,000 deaths from HBV-related liver disease or hepatocellular carcinoma (HCC), and HBV is responsible for 50% of HCC.^1–3^ The HBV genome contains a compact 3.2-kb genome that exists as a partially double-stranded, relaxed circular DNA (rcDNA) genome covalently linked to the HBV polymerase. After viral entry, the rcDNA entered into the nucleus and converted into fully double-stranded DNA, which is itself converted by ligation into covalently closed circular DNA (cccDNA).^4^ cccDNA is the stable form of HBV DNA that is responsible for its persistence in infected hepatocytes and transmission to progeny cells. There are four known genes encoded by HBV genome, called C, X, P, and S. The core protein is coded by gene C (HBcAg), and its start codon is preceded by an upstream in-frame AUG start codon from which the pre-core protein is produced. The early antigen (HBeAg) is produced by proteolytic processing of the pre-core protein. The DNA polymerase is encoded by gene P. Gene S is the gene that codes for the surface antigen (HBsAg). The HBsAg gene is one long open reading frame but contains three in frame “start” (ATG) codons that divide the gene into three sections, pre-S1, pre-S2, and S. Because of the multiple start codons, polypeptides of three different sizes called large (the order from surface to the inside: pre-S1, pre-S2, and S), middle (pre-S2, S), and small (S) are produced. The function of the protein coded by gene X is not fully understood but it is associated with the development of liver cancer. Moreover, the integration of HBV DNA sequences is a major source of HBsAg production during HBeAg-negative disease and contributes to viral persistence.^5^ There are ten different HBV genotypes (A to J), classified according to at least 8% difference in the full-length genome sequence.^6^

Vaccination is the basic and effective measure to prevent HBV infection. Safe and effective vaccines have been available since 1982. World Health Organization (WHO) has developed recommendations to ensure the quality, safety, and efficacy of recombinant hepatitis B vaccines. Therapeutic vaccination also has been used to overcome immune tolerance in chronic hepatitis B.^7^ These measures efficiently contributed to the control of hepatitis B. However, there are still a large number of people living with hepatitis B due to existing cases of infection. The current standard of care for treatment of chronic HBV infection is a daily oral dose of nucleos[t]ides analogs (e.g., lamivudine, telbivudine, adefovir, entecavir (ETV), tenofovir disoproxil (TDF), and tenofovir alafenamide (TAF); “NUCs”) or a regimen of interferon or PEGylated interferon injections 1–7 times weekly for a year.^2,5,8^ This treatment aims to reduce viral load, transaminases, and complications of liver disease. Pegylated interferon therapy can be completed in 48 weeks and is not associated with the development of resistance; however, its use is limited by poor tolerability and adverse effects such as bone marrow suppression and exacerbation of existing neuropsychiatric symptoms such as depression. Newer agents (ETV, TDF, and TAF), as the current first-line treatment, may be associated with a much lower risk of drug resistance compared with older agents (lamivudine and adefovir). However, the cure (defined as hepatitis B surface antigen loss with undetectable HBV DNA) rates after treatment remain low (3–7% with pegylated interferon and 1–12% with nucleos[t]ide analog therapy).^2,5^

Because HBeAg and HBsAg are thought to induce T cell tolerance and T cell exhaustion, which contribute to viral persistence, developing novel HBV curative regimens are extremely anxious. RNAi therapeutic is thought to be an effective modality that can realize “functional cure” for HBV treatment.^5^ The prevailing definition of “functional cure” at the present time is loss of HBsAg with or without seroconversion to anti-HBs antibodies in the blood. This is believed to be an effective endpoint of resolved acute HBV or of antiviral treatment in chronic HBV where antiviral treatment can be stopped.

RNA interference (RNAi) is a fundamental pathway in eukaryotic cells by which small interfering RNA (siRNA) is able to mediate targeted mRNA transcript cleavage, repress gene expression, and compromise gene function within living cells.^9–11^ Given the ability to knock down, in essence, any gene of interest, RNAi via siRNAs has generated a great deal of interest in both basic research and clinical application.^12,13^ siRNA-based therapeutics have shown promising prospects for treating genetic diseases, metabolic diseases, virus-infection diseases, ophthalmic diseases, age-related macular degeneration (AMD), diabetic macular edema (DME), and various solid tumors.^9,10,14^ However, developing efficient and safe delivery materials always is the most challenging but ineluctable mission siRNA therapeutic development in the field.^9,10,13^ Consequently, various delivery platforms based on either biological agents (such as exosome, peptide, antibody, aptamer, etc.),^15–20^ or chemical materials (such as liposome, polymer, dendrimer, inorganic nanoparticles, ligand-siRNA conjugate, etc.),^21–30^ or physical methods (such as electroporation, poking, etc.)^31,32^ have been established preclinically and clinically.^9,33^ Onpattro and givlarri, the first two marketed RNAi therapeutics, employed Dlin-MC3-DMA-based liposome and N-acetylgalactosamine (GalNAc)-siRNA conjugate as their delivery materials, respectively. While establishing free-to-operate proprietary delivery technology is the precondition for developing RNAi drugs in house.

In this study, a novel ionizable lipid-like material (lipidoid), LC8, was developed and thoroughly investigated for siRNA delivery to the hepatocytes. LC8, together with DPPE-mPEG_2000_ (16:0 PEG_2000_ PE), and cholesterol can form regular lipid nanoparticles, termed RBP131 in this study. siRNAs targeting apolipoprotein B (APOB) or HBV gene were employed to evaluate the delivery efficiency of RBP131. In particular, a lead anti-HBV siRNA sequence (SR16-X2M2, siHBV) with chemical modification was formulated with RBP131, to prepare RB-HBV008. The preclinical efficacy and safety performances of RB-HBV008 in multiple murine disease models and healthy animals were comprehensively investigated. It provided a novel hepatic nucleic acid delivery system, and a potential HBV-curing siRNA therapeutics.

## Results

### Formulation preparation and characterization

**Fig. 1:**
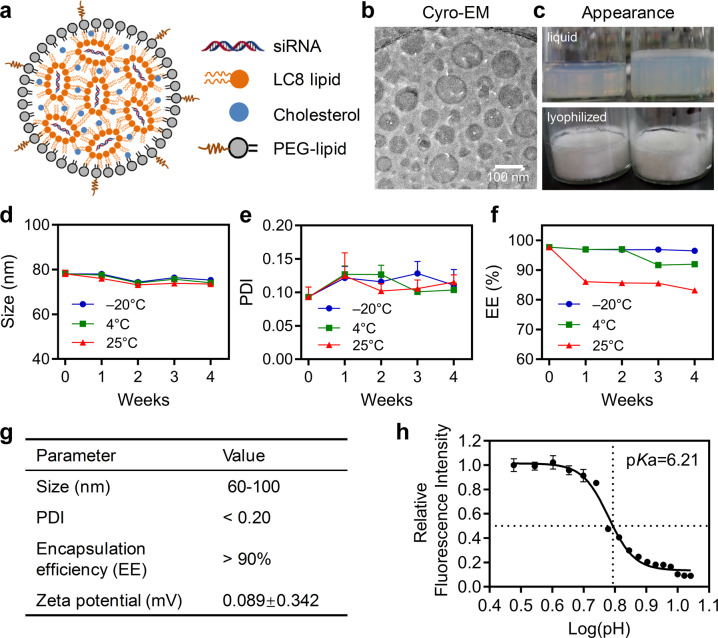
Characteristics of RBP131/siRNA nanoparticles. **a** Schematic illustration of RBP131/siRNA nanoparticle and the compositions. **b** Cryo-EM image of RBP131/siRNA nanoparticles. **c** Appearance of liquid and lyophilized RBP131/siRNA formulations. **d**–**f** Particle size (**d**), polydispersity index (PDI) (**e**), and encapsulation efficiency (**f**) changes when RBP131/siRNA was kept at −20 °C, 4 °C, 25 °C for 1, 2, 3 and 4 weeks, respectively. **g** Primary quality specifications of RBP131/siRNA formulation. **h** p*K*a titration of RBP131/siRNA complex

RBP131/siRNA complexes (Fig. 1a) were prepared by a two-step mixing method (for preparing in small scale) or a KrosFlo^®^ Research IIi Tangential Flow Filtration System (for preparing in large scale), as described in the section of method and materials. Briefly, LC8, the key ionizable lipid, together with DPPE-mPEG2000 (16:0 PEG2000 PE), cholesterol, and siRNA were mixed at indicated molar ratio and mass ratio. LC8 was synthesized by Suzhou Ribo Life Science Co. Ltd, whose chemical structure was shown in patent literature^34^. The morphology was observed with Cryo-EM, indicating that RBP131/siRNA complexes were composed of spherical vesicles with bilamellar and multilamellar structures (Fig. 1b). Drug transport and preservation are crucial for clinical drug application. Therefore, we established a state-of-the-art freeze-drying technology for RBP131/siRNA liposomal formulation. The appearance, encapsulation efficiency (EE), particle size, polydispersity index (PDI), and zeta potential were analyzed for both liquid formulation (0 weeks) and lyophilized formulations that stored at −20 °C, 4 °C or 25 °C, and reconstituted at 1 week, 2 weeks, 3 weeks and 4 weeks, respectively (Fig. 1c–f). As a result, liquid formulation looks in white or pale yellow, and the lyophilized formulation was white powder (Fig. 1c). The appearance of reconstituted lyophilized formulation is the same as the liquid formulation. Dynamic light scattering (DLS) assay revealed that the size and PDI of RBP131/siRNA were stable at all three temperatures (Fig. 1d, e). The encapsulation efficiency of RBP131/siRNA was stable at −20 °C, higher than 90% at 4 °C, higher than 80% at 25 °C for four weeks, as recorded by a modified Quant-iT Ribogreen assay (Fig. 1f). The zeta potential of this nanoparticle is almost neutral (0.089 mV) in PBS buffer at neutral pH (Fig. 1g). Generally, the size of RBP131/siRNA nanoparticles ranged from 60 to 100 nm, with a mean value of ~75 nm. The PDI is <0.2, and >90% siRNAs were entrapped by RBP131 when we kept the formulation at 4 °C even for four weeks (Fig. 1g).

It has been demonstrated that the apparent acid dissociation constant (pKa) of the ionizable amino lipids present in the lipid nanoparticles (LNPs) is a pivotal factor that determines the ability of the LNPs to deliver siRNAs into the targeted cells for eliciting gene silencing in vivo.^35,36^ The ionizable cationic lipids with pKas ranging between 6.0 and 6.5 can efficiently formulate nucleic acids at low pH and maintaining a neutral or low cationic surface charge density at pH 7.4.^37^ As a result, LNPs composed of ionizable lipids showed minimal positive charge in circulation, which can reduce disruption of plasma membranes. However, they will be ionized in an acidic environment of the endosome and lysosome, which will activate the membrane-destabilizing property of the LNP and facilitate siRNA escape from endosome and lysosome. The lipids used in this study were rationally designed. TNS assay revealed that the pKa of RBP131 is ~6.21, which perfectly met the pKa criteria (Fig. 1h).

### Distribution of RBP131/siRNA nanoparticles in vivo

Biodistribution of RBP131/siRNA nanoparticles was investigated by in vivo fluorescence imaging. RBP131/siRNA formulation was intravenously injected into the mice at the dose of 1 mg/kg. Data showed that RBP131/siRNA complexes predominantly accumulated in the liver, the kidneys, and several glands, including the submandibular gland and pancreas (Supplementary Fig. S1). A common property for these tissues is that the capillary in these tissues is fenestrated, which can facilitate them accumulating there^38^. Furthermore, cryosection observation with Confocal was performed to explore the subcellular distribution in the liver. Data revealed that Cy5-labeled siRNAs encapsulated by RBP131 dispersed on liver section (Supplementary Fig. S2). In contrast, both images of isolated organs and cryosections displayed that naked siRNA exhibited no or much weaker signal intensity in the liver (Supplementary Figs. S1, S2).

### Liver-targeted delivery efficiency of RBP131

To assess the ability of RBP131 to deliver siRNA and knockdown target gene expression in vivo, siRNA targeting apolipoprotein B (apo B) (siApoB) was first employed. ApoB is the primary apolipoprotein of low-density lipoproteins (LDL), which is a target of mipomersen (KYNAMRO^®^),^39,40^ a commercialized antisense oligonucleotide for the treatment of homozygous familial hypercholesterolemia. siApoB encapsulated by RBP131 (RBP131/siApoB) was intravenously injected into C57BL/6 mice. Livers from injected mice were harvested 72 h after injection and assayed for apoB mRNA levels by using reverse transcriptase quantitative PCR (RT-qPCR). It was shown that mice treated with RBP131/siApoB significantly reduced apoB mRNA expression, as well as the levels of TG (triglycerides) and CHO (cholesterol) in circulation (*n* = 7, *p* < 0.001) (Supplementary Fig. S3a–c). Dlin-MC3-DMA (MC3) is an ionizable lipid that has shown excellent siRNA delivery efficiency in vivo and has been approved for clinical use because it is one of the components of Onpattro.^9,35,41^ It was included as a control in this study, which revealed that MC3-based liposome and RBP131 showed comparable delivery efficacy in vivo (Supplementary Fig. S3a–c).

### Dose-response and duration profiles of RBP131/siApoB nanoparticles

**Fig. 2:**
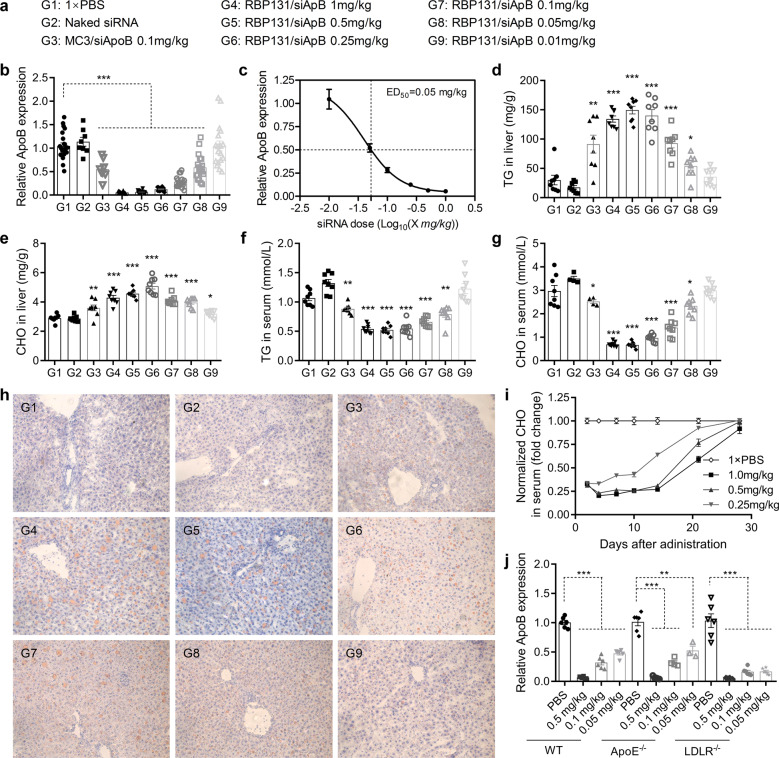
In vivo silencing performances and mechanism exploration of RBP131/siApoB nanoparticle. **a** Grouping information. **b** Relative apoB mRNA expression of the mice received various doses of the formulations. siApoB encapsulated with Dlin-MC3-DMA was included as a positive control (PosCtl). **c** ApoB mRNA expression level fitted with the function of ‘Y = Bottom + (Top-Bottom) / (1 + 10^((LogIC50-X) × HillSlope))’, with which the ED_50_ was calculated. **d**, **e** Total triglyceride (TG) (**d**) and total cholesterol (CHO) (**e**) levels in the liver. **f**, **g** TG (**f**) and CHO (**g**) levels in the serum. **h** Oil Red O staining for the liver sections. All images were enlarged 200 times with a microscope. **i** Longevity cholesterol reduction of mice receiving a single dose of RBP131/siApoB formulation. **j** Gene expression of ApoB in wild type C57BL/6 mice, apoE knockout mice (ApoE^−/−^), and LDLR knockout mice (LDLR^−/−^). Data were shown with the mean ± SEM. *P < 0.05, **P < 0.01, ***P < 0.001, vs the PBS group

To evaluate the dose-response effect of RBP131/siRNA on mediating gene silencing in vivo, siApoB was encapsulated with RBP131 and intravenously injected into C57BL/6 at doses of 1, 0.5, 0.25, 0.1, 0.05 and 0.01 mg/kg (Fig. 2a). Seventy-two hours post-injection, livers were harvested and the mRNA expression level of apoB was determined by RT-qPCR. Data revealed that apoB’s expression was suppressed dose-dependently. Knockdown efficiency higher than 90% was achieved when the dose of siRNA was higher than 0.5 mg/kg (Fig. 2b). ED_50_ was as low as 0.05 mg/kg as calculated with GraphPad Prism software (Fig. 2c). This proved that siRNA was efficiently delivered into hepatocytes and RNAi pathway was also initiated by RBP131 delivery system. In line with apoB’s expression pattern, TG (triglycerides) and CHO (cholesterol) in the liver were dose-dependently elevated (Fig. 2d, e), and their levels in circulation were reduced significantly (Fig. 2f, g). Furthermore, Oil Red O staining was performed for liver cryosections prepared with the liver tissues isolated from the animals. It was shown that lipids accumulated in the liver significantly. A higher dose of siRNA was applied, more lipids were presented in liver sections (Fig. 2h).

We further performed a time-course experiment to investigate the duration of apoB knockdown and cholesterol reduction in mice after injection of a single dose of RBP131/siApoB. Here, liposome-loaded siApoB was dosed at 1.0, 0.5 and 0.25 mg/kg, respectively. Consistent with previous observations, applying of RBP131/siApoB at 1.0, 0.5, and 0.25 mg/kg triggered robust and dose-dependent gene silencing in the mice. The total serum cholesterol (CHO) was downregulated by ~80%, ~77%, ~67% for the doses of 1.0, 0.5 and 0.25 mg/kg on day 4 post-injection, respectively (Fig. 2i). These inhibition patterns can be well maintained for 4 weeks, as they displayed a gradual retrieve course from the bottom inhibition level to the control level. These results indicated that sustained gene knockdown and phenotype could be achieved after using a single dose of RBP31/siRNA formulation, which can support biweekly, even monthly dosing regimens.

### Mechanism exploration for RBP131 mediated siRNA transportation in vivo

Previous studies have been reported that apolipoprotein E (apoE), which plays a crucial role in the clearance and hepatocellular uptake of physiological lipoproteins, may act as an endogenous targeting ligand for ionizable lipid nanoparticles (iLNPs), but not cationic lipid nanoparticles (cLNPs).^42^ Low-density lipoprotein receptor (LDLR), one of the major hepatic receptors for apoE in vivo, may also play an important role in mediating iLNPs uptake by hepatocytes, but not cLNPs.^42^ However, not all iLNPs simultaneously employ apoE and LDLR. It was shown that the efficacy of cKKE12, a lipid-like material, is dependent on apoE, but independent of LDLR.^43^ Hence, we investigated the impact of apoE and LDLR on RBP131-mediated siRNA delivery in vivo using both apoE knockout (apoE^−/−^) mice and LDLR knockout (LDLR^−/−^) mice. Here, a single injection of RBP131/siApoB at doses of 0.5, 0.1, and 0.05 mg/kg was applied to wild-type mice of C57BL/6, apoE^−/−^ mice, and LDLR^−/−^ mice, respectively. Gene expression was analyzed at 72 h post-injection. It was observed that ApoB mRNA expression was significantly inhibited in all three mice strains at all three doses, suggesting that the efficacy of RBP131 is independent of both apoE and LDLR (Fig. 2j). In consideration of that, the liver is rich in hepatic sinusoids and the capillaries of the liver is fenestrated and discontinuous,^38,44,45^ as well as that nanoparticles with a certain size range, typically smaller than 150 nm, can penetrate the endothelium system and attach hepatocytes, RBP131/siRNA LNPs is speculated to accumulate in the liver and are taken by hepatocytes via harnessing the natural physiological properties of the liver tissue.

### Characterization of lead siRNA sequence targeting HBV genes

**Fig. 3:**
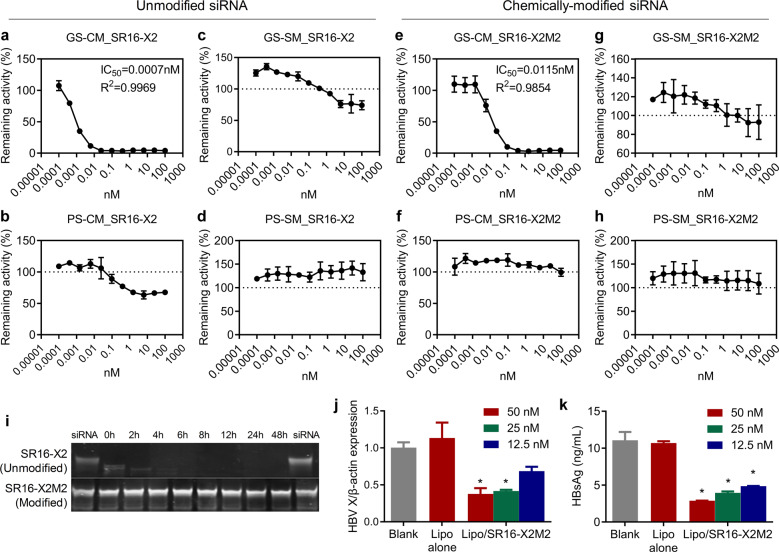
In vitro performances of anti-HBV siRNAs. **a**–**h** On target and off-target activities of the unmodified (**a**–**d**) and chemically modified (**e**–**h**) anti-HBV siRNAs. psiCHECK™ dual-luciferase reporter system, an extremely sensitive activity evaluation system, was employed in this assay. The transfection concentrations were 100, 25, 6.25, 1.5625, 0.3906, 0.0977, 0.0244, 0.0061, 0.0015, 0.0004, 0.0001 nM, respectively. GS-CM or PS-CM mean the guide strand (GS) or passenger strand (PS) of the siRNA match with its targeting mRNA incomplete match (CM) manner. The guide strand is the desired gene silencing modulator, revealing siRNA’s on-target activity. GS-SM or PS-SM means the guide or passenger strand of siRNA works via seed-region-matching, a miRNA-like pathway. Hence, gene silencing mediated by PS-CM, GS-SM, or PS-SM represents siRNA’s off-target effect. **i** Serum stability of unmodified SR16-X2 and modified SR16-X2M2 (siHBV). **j**, **k** In vitro anti-HBV activity of SR16-X2M2. Virus RNA expression (**j**) and HBsAg level in culture medium (**k**) of HepG2.2.15 receiving lipo-transfection of SR16-X2M2 at 50, 25, and 12.5 nM, respectively

RNAi therapeutic constitutes a promising therapeutic modality that may achieve e functional cure for the treatment of hepatitis B, because current nucleos(t)ides analogs barely can reduce the expression of HBV antigens that are involved in immune tolerance and T cell exhaustion. In this study, a siRNA targeting multiple transcripts of HBV genes, called SR16-X2M2 or siHBV, was selected (Fig. 3). The siRNA is chemically modified at desired sites of both strands of siRNA with a 2’-O-methyl group (2’-OMe) or 2’-Fluoro (2’-F).^34^ psiCHECK™ dual-luciferase reporter system^21^ was employed to evaluate the on-target and off-target effects of unmodified siRNA (SR16-X2) and chemically-modified version (SR16-X2M2) (Fig. 3a–h). It was observed that the IC_50_s were 0.0007 and 0.0115 nM for unmodified SR16-X2 and chemically-modified SR16-X2M2, respectively (Fig. 3a, e). Moderate silencing activity was recorded for “PS-CM_SR16-X2” at relatively high dose (>0.3906 nM) (Fig. 3b), suggesting that the passenger (sense) strand of SR16-X2 showed moderate silencing activity if certain transcript was completely complementary with the passenger strand. In addition, slight silencing activity was also exhibited for “GS-SM_SR16-X2” (Fig. 3c), which means the guide (antisense) strand of SR16-X2 potentially inhibited target expression if the target sequence was complementary with the seed region of the guide strand. However, these undesired downregulations of luciferase activity were not observed for SR16-X2M2, the chemically-modified version of SR16-X2 (Fig. 3f, g). This study strongly demonstrated that potential off-target silencing activity, although it only occurred at high transfection concentration, was completely erased by rationally positioning chemical modifications on both the sense and the antisense strands of siRNA.

In addition, with chemical modifications, the RNase resistance capability of SR16-X2M2 was also significantly enhanced (Fig. 3i). In addition, the siHBV was transfected to HepG2.2.15, a human hepatoma cell line that has several copies of the HBV genome inserted into its own genome, with lipofectamine 2000. As a result, when the transfection concentrations were 50 nM, 25 nM and 12.5 nM, the expressions of X gene mRNA were reduced 62.3% (*P* < 0.05), 58.3% (*P* < 0.05), 31.6%, respectively; and the HBsAg expressions were also decreased 74.0% (*P* < 0.05), 64.4% (*P* < 0.05), 56.2% (*P* < 0.05), respectively (Fig. 3j, k).

### Gene silence and mechanism validation in a partial gene-integrated transgenic mouse model (model 1)

HBV transgenic mouse model, C57BL/6J-TgN (AlblHBV) 44Bri/J,^46^ was employed to investigate the anti-virus effect of RBP131-loaded siHBV (RB-HBV008). 1 × PBS, naked siHBV, MC3 liposome-loaded siHBV, empty RBP131 nanoparticles, and RBP131-loaded scramble siRNA (negative control siRNA, NC siRNA) were included as controls. siRNA was dosed at 1 mg/kg, and the mRNA level of X gene was quantified at 72 h post treatment. MC3/siHBV and RB-HBV008 significantly repressed the expression of X gene (*P* < 0.005 for both groups, vs 1 × PBS), as 95.2% and 94.4% knockdown efficiency was observed, respectively (Supplementary Fig. S4). The silencing efficiency shown by anti-HBV siRNA is comparable with that shown by anti-apoB siRNA. In contrast, other four groups displayed no gene silencing. In addition, RNAi mechanism was validated in this model by using RACE (rapid amplification of cDNA ends) PCR assay. Data clearly showed that the transcript of HBV gene was cleaved at position 10 from the 5’ end of the antisense strand (Supplementary Fig. S5). These results illustrated that RBP131 could efficiently deliver anti-HBV siRNA into hepatocytes in animal disease model and repress targeted gene expression via RNAi pathway, encouraging us to perform more assays to investigate the treatment performances of RB-HBV008 in more animal models.

### Reduction of HBsAg and HBV mRNA in transgenic mouse model with 1.0 copy of HBV genome (model 2)

In order to evaluate the anti-HBV activity of RB-HBV008, the second HBV transgenic mouse model, C57BL/6J-M-Tg (HBV C1.0), was employed. This model was established by J. Ren and colleagues from Fudan University,^47^ and kept by Shanghai Biomodel Organism Science & Technology Development Co.,Ltd. A single copy (3.2 kb-length) linearized HBV genome (C1-type) was integrated into mouse chromosome 9 in the second exon of an unknown gene AI604832. HBsAg is positively expressed in the serum, the liver, and the kidneys. HBcAg can also be positively detected in the liver and the kidneys. However, HBsAb, HBeAg, HBeAb, HBcAb, and HBV DNA are all negatively expressed in circulation. It cannot produce the HBV virus particle since the genome was cut at the position of 1806.

**Fig. 4:**
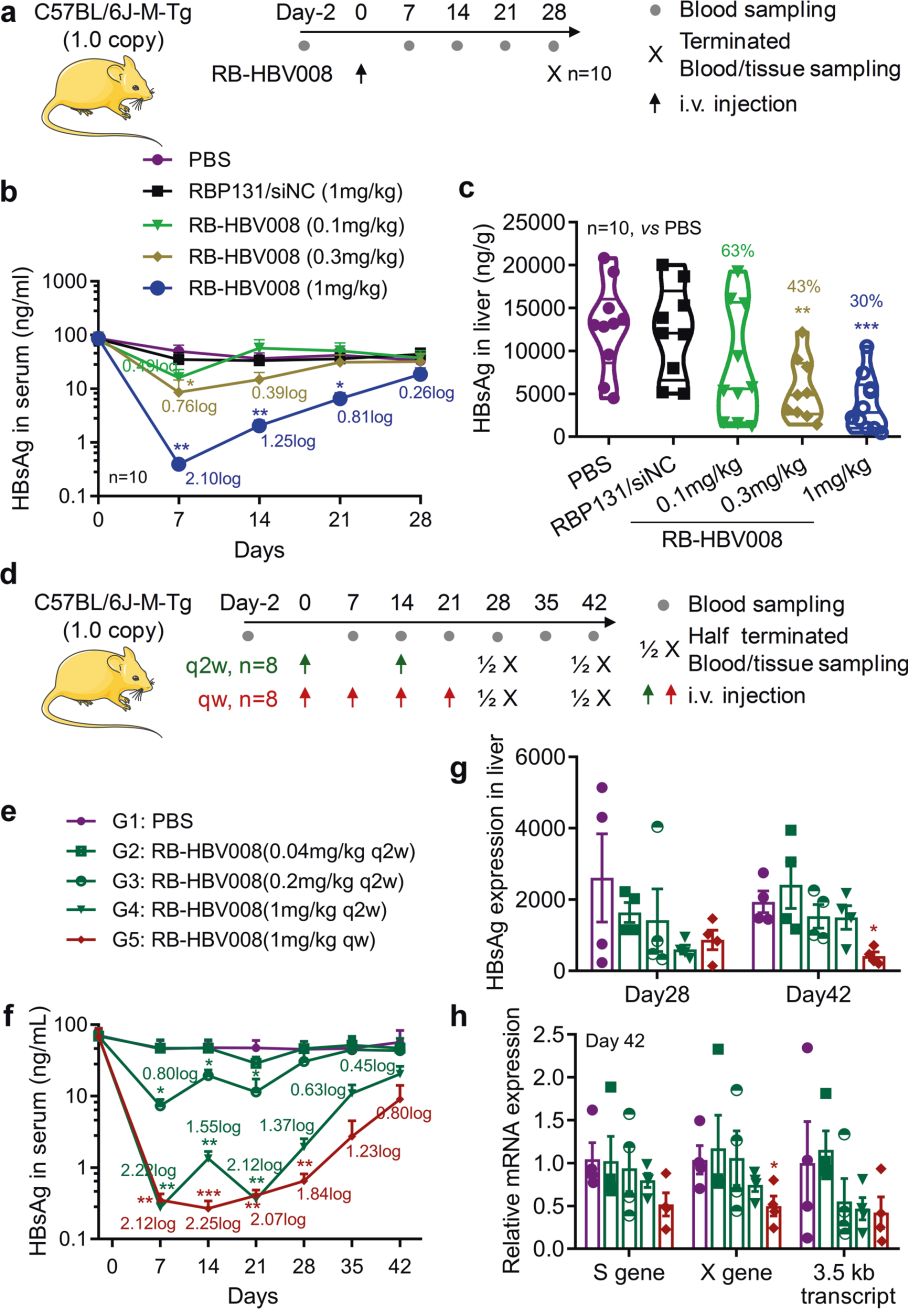
Efficacy of RB-HBV008 in C57BL/6J-M-Tg (HBV C1.0) mouse model. **a** Treatment schedule of “1.0M-SD” study. **b** Level of HBsAg in serum during the treatment course. **c** Expression of HBsAg in the liver tissues was analyzed at day 28. **d** Treatment and sampling schedule of “1.0M-MD1” study. **e** Grouping information. **f** HBsAg level in the circulation during the treatment course. **g** HBsAg expression in the liver tissue on day 28 and day 42. **h** Relative mRNA expressions of the X gene, S gene, and 3.5 kb transcript on day 42. Data were shown as mean ± SEM. *P < 0.05; **P < 0.01, ***P < 0.001 (two-tailed *t* test)

Three studies were performed with this model. First, a signal dose of RB-HBV008 was intravenously injected into the mice at the doses of 0.1, 0.3, and 1.0 mg/kg, respectively (Fig. 4a–c, study termed “1.0M-SD”). RBP131/siNC and PBS were included as negative controls. Blood samples were collected every week, and the assay was terminated on day 28 (four weeks after administration) (Fig. 4a). Data revealed that the level of S antigen of HBV (HBsAg) in circulation was significantly inhibited in the animals receiving RB-HBV008 treatment. Over two log (2.10 log, or 99.2%, *P* < 0.01) reduction was achieved in the animals dosed at 1.0 mg/kg, and the repression was well maintained for four weeks because HBsAg level still decreased 0.26 log (44.5%) compared to that of PBS group at day 28 (Fig. 4b). Moreover, the expression of HBsAg in the liver tissue was also repressed dose-dependently (Fig. 4c).

Second, multiple doses of RB-HBV008 were injected into the mice weekly (four doses in total) or biweekly (two doses in total) (Fig. 4d–h, study termed “1.0M-MD1”). For the biweekly-dosed mice, siRNA was dosed at 0.04, 0.2, and 1.0 mg/kg, respectively. While for a weekly-dosed group, siRNA was dosed at 1.0 mg/kg. It was observed that HBsAg in serum was significantly downregulated dose-dependently and time-dependently (Fig. 4d–f). Over 99% (>2 log, *P* < 0.01) knockdown could be achieved on day 7 post administration for biweekly treating mice. The inhibition efficiency of HBsAg expression was maintained at 64% after four weeks from the last dose (Fig. 4e, f). Moreover, the HBsAg level was downregulated over 99% (>2 log, *P* < 0.01) during the weekly dosing course, and 84% repression efficiency could be observed after three weeks from the last dose (Fig. 4e, f). HBsAg expressions in the liver tissue on day 28 and day 42 were also down-regulated dose-dependently (Fig. 4g). In addition, the expressions of viral RNAs, including X gene, S gene and the 3.5 kb transcript, were examined on day 42, four weeks after the last dose of “biweekly-dosing groups” and three weeks after the last dose of the “weekly-dosing group”. Data showed that the expressions of these three transcripts were dose-dependently inhibited on day 42 (Fig. 4h). Approximate 50% gene silencing was observed for the group of mice dosed at 1 mg/kg on day 42, three weeks after the last dose. Therefore, RBP131-loaded ant-HBV siRNA significantly inhibited the expression of HBsAg and viral mRNAs in a dose and time dual-dependent manner.

Third, four doses of RB-HBV008 were biweekly administered to the mice at the doses of 0.1, 0.3, 0.6 and 1.0 mg/kg, respectively (Supplementary Fig. S6, study termed “1.0M-MD2”). Data revealed that HBsAg also was significantly downregulated dose-dependently (Supplementary Fig. S6). The inhibition was maintained for four weeks after the last dose, in line with the observations of the first and the second experiments. These data potentially support the biweekly use of RB-HBV008 in clinical practice.

### Anti-HBV activity of RB-HBV008 in mouse model with 1.3 copy of HBV genome (model 3)

**Fig. 5:**
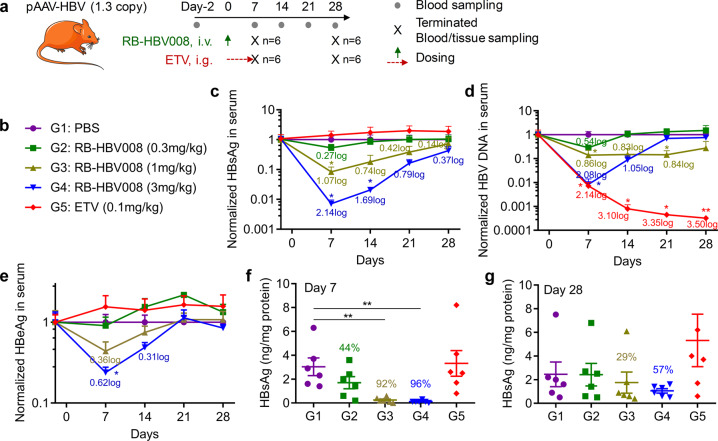
Efficacy of RB-HBV008 in pAAV-HBV mouse model. **a** Treatment and sampling schedule of the study. **b** Grouping information. **c**–**e** Levels of HBsAg (**c**), viral DNA (**d**), and HBeAg (**e**) in circulation during the treatment course. **f**, **g** Expressions of HBsAg in the liver tissue were examined on day 7 (**f**) and day 28 (**g**). Data were shown as mean ± SEM. *P < 0.05, **P < 0.01 (two-tailed *t* test)

To further assess the anti-virus activity of RB-HBV008, the third HBV animal model was used (Fig. 5a). This model, termed “pAAV-HBV”, was established by hydrodynamic injection of pAAV-HBV1.3 plasmid (10 μg/mouse) that contains a 1.3-fold-overlength genome of HBV.^48^ This model expresses all elements of HBV virus, including HBsAg, HBeAg, HBV DNA, et al., however, it still has some limitations on HBV lifecycle, such as a low level of HBeAg and lack of cccDNA. Data demonstrated that single administration of RB-HBV008 at 0.3, 1, and 3 mg/kg triggered robust and dose-dependent inhibition of HBsAg expression with 46.9%, 91.6% (1.07 log, *P* < 0.05), 99.3% (2.14 log, *P* < 0.05) knockdown, respectively. The inhibition efficiencies were maintained at 28.0% and 56.9% on day 28 for the groups dosed at 1 and 3 mg/kg, respectively (Fig. 5b, c).

Entecavir (ETV), a first-line antiviral medication used in the treatment of HBV infection, was included as a positive control because this model expresses HBV DNA. Entecavir reduces the amount of HBV in the blood by reducing its ability to multiply and infect new cells. But limited, even no, reduction of antigen protein of HBV can be achieved using ETV. In line with our expectation, it failed to trigger HBsAg reduction during the treatment course (Fig. 5c). However, HBV DNA was significantly downregulated by ETV, even reached the lowest detection limit on days 21 and 28 (Fig. 5b, d). Meanwhile, RBP131 loaded anti-HBV siRNA also leaded dose-dependent decrease of HBV DNA, with 72.4% (0.54 log), 86.1% (0.86 log, *P* < 0.05) and 99.2% (2.08 log, *P* < 0.05) inhibition efficiency, respectively. Moreover, 56.2% (0.36 log) and 76.1% (0.62 log, *P* < 0.05) downregulation of HBeAg also could be achieved when RB-HBV008 were dosed at 1 and 3 mg/kg, respectively (Fig. 5b, e). The expression of HBsAg in the liver was also dramatically repressed both on day 7 and day 28 post-administration (Fig. 5f, g). Taken together, RB-HBV008 not only significantly inhibited the expression of HBsAg, but it could also block the synthesis of HBeAg and DNA replication. HBeAg and HBsAg play important roles in chronic infection.^49^ HBeAg is thought to induce T cell tolerance to HBeAg and core, which may contribute to viral persistence.^50^ High HBsAg production is believed to contribute to T cell exhaustion, resulting in limited or weak T cell responses and even deletion of T cells recognizing specific epitopes.^51^ In addition to enveloping the virion, HBsAg forms as many as 100,000 times more subviral particles than virions.^52^ For CHB patients, the desired endpoint of treatment is seroclearance of HBsAg, referred to as a “functional cure”, resulting in improved long-term prognosis.^53^ HBsAg loss is considered a hallmark of effective immune control of HBV.^54^ The drawback of ETV clinical application is it can only stimulate limited antigen response for Hepatitis B treatment. These data demonstrate that siRNA, as a potential “functional cure” agent, constitutes a novel and powerful HBV treatment strategy by either alone application or combined with ETV (or other nucleot(s)ide analogs).

### Toxicity evaluations in normal animals

**Fig. 6:**
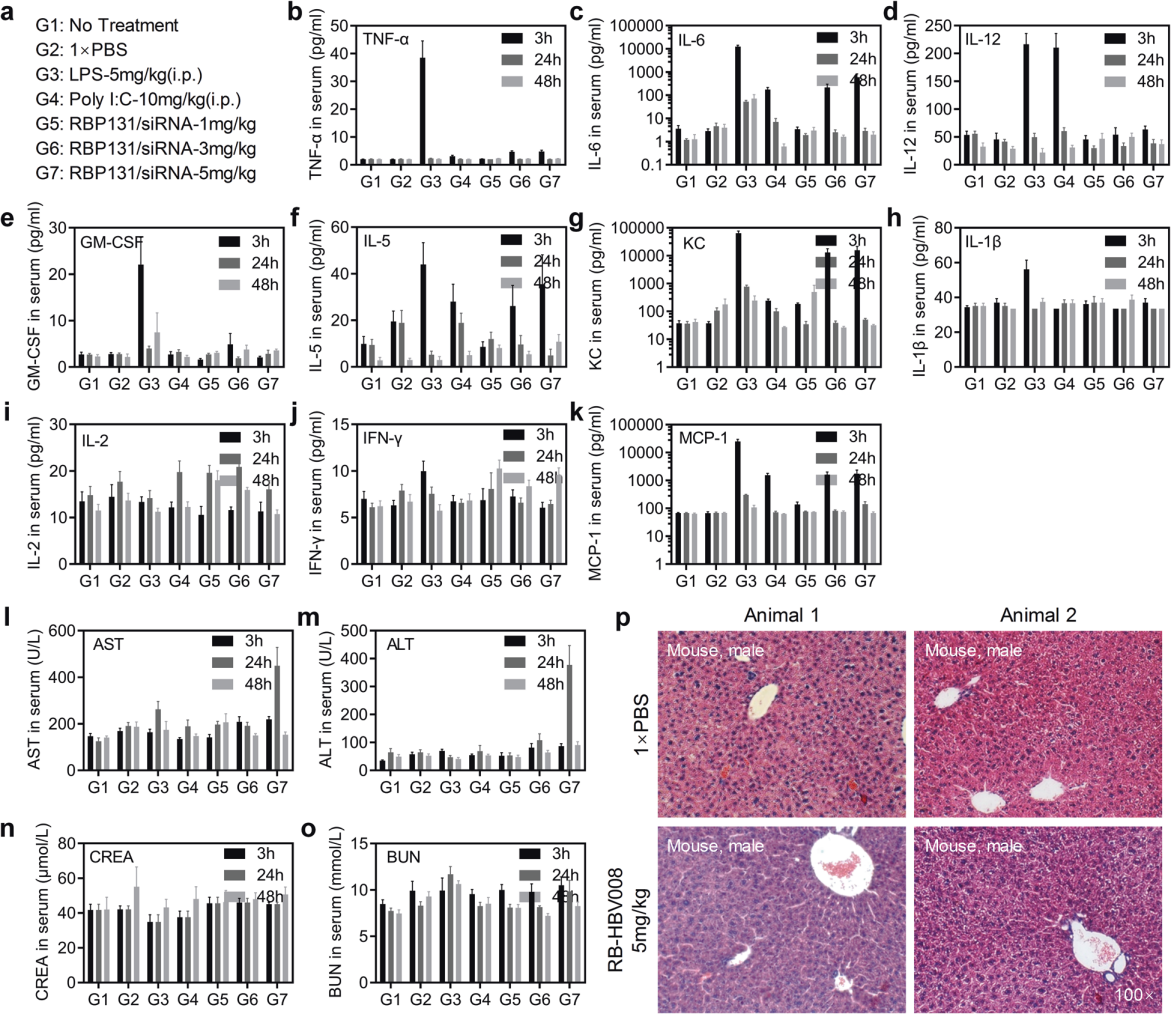
Toxicity evaluation of RB-HBV008 in mice. **a** High doses (1.0, 3.0, and 5.0 mg/kg) of RBP131/siRNA formulations were intravenously injected into the CD-1 mice. Two negative controls (animals without any treatment (G1) and PBS (G2)) and two positive controls (Lipopolysaccharides (LPS, G3) and poly I: C (G4)) were included in the assay. **b**–**k** Cytokine concentrations in serums were collected at 3, 24, and 48 h post-administration. Ten cytokines including TNF-α, IFN-γ, IL-1β, IL-2, IL-5, IL-6, IL-12(p70), MCP-1, KC, GM-CSF were analyzed with Luminex^®^ assay. **l**–**o** Levels of AST, ALT, CREA, and BUN in serums of the mice received various treatments at 3, 24, and 48 h. **p** Histological analysis of liver tissue sections prepared from the mice receiving the treatments of PBS or RB-HBV008 at 5.0 mg/kg. Data were shown as mean ± SEM

In addition to efficacy, the clinical use of RNA therapeutics requires a favorable safety profile. Here we performed complicated safety evaluations in mice and rats. First, a single dose toxicity study of RBP131/siRNA nanoparticles was conducted, followed by a serum cytokine panel, liver blood chemistry, and histopathology in male CD-1 mice dosed at 1, 3, and 5 mg/kg siRNA (Fig. 6). Lipopolysaccharide (LPS) and poly I:C were included as positive controls. They were intraperitoneally injected at the dose of 5 and 10 mg/kg, respectively. Blood samples were collected at 3, 24, and 48 h after injection, and livers were harvested at 48 h after injection. Cytokine inducement assays (Fig. 6a–k) revealed that a transient increase in expressions of IL-5, KC (CXCL1), and MCP-1 at 3 h in the mice received 3 and 5 mg/kg of RBP131/siRNA, compared with 1 × PBS-treated mice, and this increase returned to baseline within 24 h (Fig. 6f, g, k). However, LPS induced a significant increase of many cytokines, such as TNF-α, IFN-γ, KC, MCP-1, GM-CSF, IL-6, and IL-12 at 3 h post-injection. LPS also triggered a remarkable decrease in body weights and enhancement of organ coefficient of the spleen (the weight of the spleen was divided by the body weight) (Supplementary Fig. S7). Poly I: C induced remarkable elevation of IL-12 at 3 h post-injection (Fig. 6d).

Furthermore, we analyzed serum chemistry for aspartate transaminase (AST), alanine transaminase (ALT), CREA (serum creatinine), BUN (blood urea nitrogen) of RBP131/siRNA-treated mice in comparison to controls (Fig. 6l–p). In line with the observations of cytokine inducement, AST and ALT were transiently elevated at 3 h after injection, and it recovered to normal level within 48 h, for the doses of 3 and 5 mg/kg (Fig. 6l, m). However, no significant differences for CREA and BUN were observed at all three observation time points (Fig. 6n, o). Finally, histopathology analysis was performed for the liver tissues harvested at 48 h after injection, and no significant histological abnormality was observed for RBP131/siRNA-treated mice (Fig. 6p). Taken together, this preliminary single-dose toxicity study supports a conclusion that RBP131/siRNA nanoparticles are well tolerated in mice at these doses.

Moreover, a toxicity study in SD rats was also performed. Data revealed that the animals can well tolerate the challenge of both single-dose and multiple doses of RB-HBV008 at the dose of 5 mg/kg. Compared with PBS group, no significant changes were observed regarding the body weight, food consumption, and organ-to-brain coefficients. Meanwhile, there is no significant difference between RB-HBV008 group and PBS group for the blood biochemistries and the histopathological staining (data not shown).

## Discussion

Chronic infection of hepatitis B virus may lead to cirrhosis or cancer, which constitutes a major threat to global public health. Current treatment agents can well control the replication of viral DNA; however, they cannot effectively reduce the expression of antigen proteins. In hepatitis B patients, antigen proteins mediate immune tolerance and cause excessive consumption of T cells. RNAi therapeutics can mechanically inhibit the expression of all viral gene transcripts, which can not only reduce the replication of viral DNA but also significantly inhibit the expression of antigen proteins, therefore it is expected to achieve functional cure in clinical treatment.

In this study, a novel ionizable lipid-like material called LC8 was developed to surmount the delivery obstacle, the most challenging issue for RNAi drug development. LC8 is a five-tailed amine-containing compound. Based on LC8, a lipid nanoparticle termed RBP131 was prepared, whose pKa value was approximate 6.21, excellently matching the pKa requirement (6.0–6.5) of liposomal delivery materials that can trigger the pH-responsive endosomal escape of siRNA during the subcellular trafficking process.^35,36^ In order to meet the need for storage and transportation during the clinical application, state-of-the-art lyophilization technology was established by adjusting detailed lyophilization conditions of the lyophilizer, and by selecting proper protecting regents (saccharose was used finally). As result, all quality control parameters of the reconstituted lyophilized formulation, such as the particle size, zeta potential, encapsulation efficiency, polydispersity index, as well as appearance are comparable to, or the same with those of the liquid formulation. More importantly, the in vivo silencing activities are also similar between these two formulations. This is quite crucial for liposomal siRNA drug development.

The ED_50_ of RBP131/siRNA was evaluated with siRNA targeting APOB, because silencing this target enables evaluating the delivery performances in multiple dimensions, including mRNA expression in hepatocytes, total triglyceride, and total cholesterol levels both in liver tissue and in circulation, as well as Oil Red O staining of the tissue sections. In addition, an association between hypercholesterolemia and increased apoB protein levels, together with the observation that reductions in apoB synthesis reduce LDLc and the incidence of atherosclerosis, has generated interest in apoB as a therapeutic target.^55^ Mipomersen (KYNAMRO^®^),^39,40^ an APOB-targeting antisense oligonucleotide, was approved by the FDA (the US Food and Drug Administration) for lowering LDL levels in patients with Homozygous Familiar Hypercholesterolemia. However, silencing APOB gene potentially elevates the lipid accumulation in the liver tissue, leading to that the FDA label of Mipomersen includes a black box warning of hepatoxicity. In our study, RBP131 exhibited extremely excellent siRNA delivery efficiency, with an ED_50_ of ~0.05 mg/kg when employing anti-APOB siRNA. This value was further reduced to 0.02 mg/kg if using siRNA targeting murine clotting factor VII (FVII) to determine the ED_50_ (data not shown), which was comparable to Dlin-MC3-DMA-based formulation^35^.

The underlying transportation mechanism was explored with ApoE knockout (ApoE^−/−^) mice and LDLR knockout (LDLR^−/−^) mice because it was reported that ionizable lipid nanoparticle (iLNP) may bind with ApoE in circulation and then cross hepatocyte membrane via LDLR-mediated endocytosis. However, our results showed that RBP131, although it was also an ionizable lipid formulation, rely on neither ApoE nor LDLR. It is speculated that RBP131/siRNA complexes mainly accumulate in the liver by taking the advantage of fenestrated sinusoidal capillary structure in the liver. Certain unknown mechanisms may also involve in this process. The exact mechanism remains to be further elucidated.

Furthermore, a siRNA targeting multiple transcripts of HBV genes was selected, and encapsulated by RBP131, to prepare a hepatitis B treatment agent termed RB-HBV008. The therapeutic effects were comprehensively investigated in three animal models, suggesting RB-HB008 could trigger dose-dependent reductions of viral RNA (X gene, S gene, and 3.5 kb transcript), viral DNA, surface antigen (HBsAg), and e antigen (HBeAg). Higher than 2 log_10_ (>99%, *P* < 0.01) decline and longer than four weeks reduction of HBsAg could be achieved after receiving a single intravenous dose of RB-HBV008, which was not recorded in ETV-treated animals. Higher than 2 log_10_ (>99%, *P* < 0.05) decrease of viral DNA and 0.6 log10 (76%, *P* < 0.05) downregulation of HBeAg were also observed in the animal model. Multiple dosing of RB-HBV008 resulted in sustained reduction of HBsAg and viral RNA. In addition, the toxicity performed in mice and rats revealed that RB-HBV008 was well tolerated by the animals at 5 mg/kg, a dose 100 times higher than ED_50_ when silencing APOB, and over 50 times higher than 0.1 mg/kg as the “Minimal Anticipated Biological Effect Level (MABEL)” for inhibiting HBV DNA and antigens.

This study has several limitations. First, all pharmacodynamics studies were performed with the murine disease model. The murine hepatocytes cannot be infected by HBV, whose genetic background, infection mechanism, and pathophysiological situation are different from clinical manifestations. Second, a comparison of ETV and siRNA was conducted in this study, but it was not evaluated that if combination use of ETV and siRNA will induce a synergistic treatment effect. Thirdly, efficacy and safety were the most critical aspects for drug development. More toxicity evaluations need to be performed according to the preclinical safety evaluation guidelines, which will improve the clinical transition feasibility.

In addition, several issues should be taken into consideration when conducting clinical studies in the future. First, whether the patients have experienced the treatments of nucleos(t)ide analog reverse-transcriptase inhibitors (NUCs), and HBeAg is positive or negative, are of great importance for the treatment outcomes when applying RNAi therapeutics^5,56^. Second, it has been demonstrated that HBsAg can be also generated from the integrated DNA, in addition to from the cccDNA. Therefore, siRNA sequence should be designed to target both free and integrated sites of HBV genome. siRNA developed in this study theoretically matches this requirement. Alternatively, a cocktail of siRNAs targeting different sites or different transcripts can also be used for anti-HBV RNAi therapeutic development. Thirdly, although Dlin-MC3-DMA-based liposomal siRNA formulation has reached clinical application along with the approval of Onpattro, enough emphasis should be paid to the safety profile of RB-HBV008, another liposomal RNAi modality. Finally, combination therapy by using siRNA, NUCs, even other modalities harnessing novel action mechanisms, constitutes a valuable choice to achieve better treatment outcomes.

In summary, this study established a proprietary hepatic siRNA delivery platform and a state-of-the-art lyophilization technology, based on which an HBV-curing RNAi formulation was thoroughly investigated preclinically in multiple disease murine models and rats, highlighting its promising development prospects in the next stage for HBV functional cure.

## Materials and methods

### Materials

All siRNA used in this study were synthesized by Suzhou Ribo Life Science Co. Ltd. (Kunshan, China). In order to enhance their stability and specificity, chemical modifications with methoxy group or fluorine at the 2’ site hydroxyl groups, or phosphorothioate at the phosphonate backbone were placed at certain sites of the sense and antisense strands of siRNA. For Cy5-labeled siRNA, the Cy5 fluorophore was placed at the 5’ end of the sense strand. TRIzol^®^ was purchased from Thermo Fisher Scientific (Eugene, OR, USA). Reverse transcription kit and real-time PCR kit (UltraSYBR Mixture) were purchased from Promega Corporation (Fitchburg, Wisconsin, USA) and Beijing ComWin Biotech Co., Ltd. (Beijing, China), respectively. RNAlater^®^ were purchased from Sigma–Aldrich (St Louis, MO, USA). DAPI (4’, 6-diamidino-2-phenylindole, for staining nuclei) was from Zhongshan Golden Bridge Biotechnology Co., Ltd., Beijing, China, and fluorescein isothiocyanate-labeled phalloidin (for staining F-actin) was from Sigma-Aldrich (MO, USA). Pentobarbital sodium was provided by Peking University Laboratory Animal Center.

### Formulation of lipid nanoparticles (LNPs)

The Lipidoid component of RBP131 was synthesized by Suzhou Ribo Life Science Co. Ltd. RBP131 formulation was prepared according to a modified protocol adopted from reference,^57^ or an example described in patent literature.^34^ Briefly, LC8, cholesterol (Sigma–Aldrich, St Louis, MO), and “16:0 PEG_2000_ PE” (MW 2749.391, Sigma–Aldrich) were prepared in ethanol and mixed to yield a molar ratio or 59:29:12. Mixed lipids were added to 200 mM sodium acetate buffer (pH 5.2) to yield a solution containing 25% ethanol, resulting in spontaneous formation of empty lipidoid nanoparticles. Then empty lipidoid nanoparticles and siRNA solution in 25% ethanol were rapidly mixed together using a peristaltic pump at ~1:15 (wt/wt) siRNA/total lipids, followed by incubating at 50 °C for approximate 20 min. Finally, ethanol removal and buffer exchange of siRNA-containing lipidoid nanoparticles was achieved by dialyzing against 1 × PBS for 2 h in 100 KD MWCO cassettes (Float-A-Lyzer^®^ G2, Spectrum Laboratories Inc. New Brunswick, New Jersey, USA). If needed, 100 KD MWCO ultrafiltration tube (Amicon^®^ Ultra-15 or Ultra-4, EMD Millipore, Billerica, Massachusetts, USA), or 1 × PBS (or normal saline) can be used to concentrate or dilute the solution to the desired concentration. Alternatively, a KrosFlo^®^ Research IIi Tangential Flow Filtration System can be also used to prepare RBP131/siRNA formulation on large scale. In addition, a lyophilization technology was established, by which the liquid RBP131/siRNA formulation can be lyophilized. Sterile water was used to reconstitute the lyophilized powder before administration.

### Detection of encapsulation efficiency

siRNA encapsulation efficiency was determined by modified RiboGreen RNA assay (Invitrogen, Carlsbad, CA).^58^ Briefly, siRNA entrapment was determined by comparing the signal of the RNA binding dye RiboGreen in formulation samples in the absence and presence of the detergent Triton-X100. In the absence of detergent, the signal comes from accessible (unentrapped) siRNA only. In the presence of detergent, the signal comes from total siRNA. In all preparations used for this study, the encapsulation efficiency was above 90%.

### Characterization of particle sizes and zeta potential

The particle sizes and zeta potentials of the RBP131/siRNA complexes were measured using a Zetasizer Nano ZS (Malvern Instruments, Inc., Worcestershire, UK) at a wavelength of 677 nm with a constant angle of 90° at room temperature. The complex was prepared according to the above-mentioned protocol. Typically, ~0.8 ml of solution was used to detect size and zeta potential.

### In situ determination of pKa using TNS

TNS (6-(p-Toluidino)-2-naphthalenesulfonic acid sodium salt) assay was used to detect the pKa value of RBP131 formulation. Empty RBP131 vesicles were prepared in 1 × PBS with a concentration of ~6 mM total lipids. TNS was prepared as a 100 μM stock solution in distilled water. RBP131 vesicles were diluted to 30 μM lipid in 2 mL of buffered solutions containing 10 mM HEPES, 10 mM MES, 10 mM ammonium acetate, 130 mM NaCl, where the pH ranged from 3.0 to 11.0. An aliquot of the TNS solution was added to give a final concentration of 1 μM and following vortex mixing fluorescence intensity was measured at room temperature in a fluorometer (Synergy™ HT, BioTek, Winooski, VT, USA) using excitation and emission wavelengths of 321 and 445 nm. A sigmoidal best fit analysis was applied to the fluorescence data and the pKa was measured as the pH gave rise to half-maximal fluorescence intensity.

### Cryogenic electron microscopy (Cryo-EM)

Cryo-EM samples were made by Vitrobot (FEI Company) at a controlled temperature (25 °C) and at saturation. Cryo-EM grids were frozen using a Vitrobot Mark IV (FEI) as follows: 6 μL of the sample was applied to a glow discharged Quantifoil R1.2/1.3 holey carbon 300 mesh gold grid. To remove excess solution and produce a thin liquid film the drop is blotted manually in the Vitrobot. The blotted sample is then plunged into liquid ethane (−183 °C) to form a vitrified specimen and transferred to liquid nitrogen (−196 °C) for storage. Cryo-EM data of vitrified specimens were recorded on a Titan Krios (FEI) operated at 300 kV, equipped with a Gatan K2 Summit camera. SerialEM was used for automated data collection.

### Cell culture

HepG2.2.15 is a widely-used cell line for studying HBV in vitro. It contains 2 head to tails stably integrated HBV of the genotype D. It was kept by Suzhou Ribo Life Science Ltd. Co. and is cultured with DMEM (Dulbecco’s Modified Eagle’s Medium) supplemented with 10% fetal bovine serum, 100 units/ml penicillin, and 100 µg/ml streptomycin at 37 °C in a humidified atmosphere of 5% CO_2_. Hek293A was used in psiCHECK™ assay, which was cultured with the same protocol of HepG2.2.15.

### Off-target effect evaluation in vitro

The off-target effect is a critical issue for RNAi therapeutic development, since off-target may trigger serious toxicity in vivo.^59,60^ siRNA may induce off-target effect via multiple mechanisms, such as (1) passenger (sense) strand of siRNA bind with undesired mRNA target in a complete-match manner, and inhibits its translation (siRNA working pathway); (2) either passenger (sense) or guide (antisense) strand of siRNA recognizes undesired target mRNA via seed-region matching (miRNA working mechanism); (3) gene disturbing resulting from immune-response stimulated by siRNA molecule. These weaknesses can be surrounded by positioning proper chemical modifications at specific sites of both the passenger and the guide strands of siRNA. Lead siRNA sequence targeting HBV genes (SR16-X2) was screened in this study, and rational chemical modifications (generating a molecule called SR16-X2M2) were used to enhance its performance. psiCHECK™ dual-luciferase reporter system (Promega, Madison, WI, USA)^21^ was employed to evaluate the on-target and off-target activity of unmodified siRNA and chemically-modified siRNA. Briefly, both firefly and renilla luciferase expression sequences were incorporated into the plasmid, and the target sequence of SR16-X2 (without modification) and SR16-X2M2 (siHBV, with modification) was inserted into the plasmid at the 3’-UTR of renilla luciferase. Then the unmodified or chemically-modified siRNAs were transfected into Hek293A cells at concentration gradients of 100, 25, 6.25, 1.5625, 0.3906, 0.0977, 0.0244, 0.0061, 0.0015, 0.0004, 0.0001 nM, together with the psiCHECK™ plasmid, by lipofectamine 2000. Twenty-four hours later, the cells were lysed with Passive Lysis Buffer (Promega), 10 μL of lysate of each treatment was transformed to 96-well plate, and the activities of both firefly and renilla luciferases were evaluated with the Dual-Luciferase Reporter Assay System (Promega). Finally, renilla luciferase activity was normalized to the firefly luciferase activity, and siRNA silencing activity was calculated by comparison with the negative control sample. The IC_50_ was calculated with GraphPad Prism 8.0 software.

### Serum stability assay

Serum is rich of RNase, therefore fetal bovine serum (FBS, Sigma) was used to evaluate the stability of RNase-resistance capability of unmodified and modified siRNA. Unmodified SR16-X2 or modified SR16-X2M2 were incubated in 10% FBS (v/v, in 1 × PBS) at 37 °C. Samples were collected and immediately frozen at −20 °C at 0, 2, 4, 6, 8, 12, 24, and 48 h post-incubation. Then all samples were diluted 2 times with DEPC water and mixed with loading buffer, followed by separating in 20% non-denaturing PAGE (polyacrylamide gel electrophoresis) for 120 min at a constant voltage of 150 V. Finally, gels were stained with Sybr Gold for 20 min, exposed by Vtlber Lourmat imaging system (Frence).

### Anti-HBV effects of SR16-X2M2 in vitro

In vitro activity of SR16-X2M2, the lead compound selected to perform preclinical evaluation was assessed with HepG2.2.15. siRNA was transfected with lipofectamine 2000 at the concentrations of 50, 25, and 12.5 nM, respectively. Twenty-four hours later, the expression of X gene and HBsAg were determined by RT-qPCR and ELISA, respectively.

### Animals

C57BL/6 mice (6–8 weeks old, 18–22 grams) were purchased from Vital River Laboratory Animal Technology Co., Ltd. (Beijing, China). ApoE^−/−^ mice (C57BL/6 background, 6–8 weeks old, 18–22 grams) and LDLR^−/−^ mice (C57BL/6 background, 6–8 weeks old, 18–22 grams) were purchased from Changzhou Cavens Laboratory animal Co., Ltd. (Changzhou, Jiangsu, China). Animals were maintained in the Peking University Laboratory Animal Center (an AAALAC-accredited and specific pathogen-free (SPF) experimental animal facility). ApoE^−/−^ and LDLR^−/−^ were ApoE (apolipoprotein E) and LDLR (low-density lipoprotein receptor) knockout mice, respectively. They originally came from Jackson Laboratories. HBV transgenic mice (C57BL/6j-TgN(AlblHBV)44Bri) used in this study were also kept in Peking University Laboratory Animal Center. In addition, efficacy of RB-HBV008 was also assessed with other two disease murine models. The first one was a transgenic mouse model, C57BL/6J-M-Tg (HBV C1.0), which was established by Fudan University.^47^ The second one was pAAV-HBV mouse model that was established by hydrodynamic injection of pAAV-HBV1.3mer plasmid DNA (10 μg/mouse) that contains a 1.3-fold-overlength genome of HBV.^48^ It was kept by WuXi AppTec, a leading global contract research outsourcing provider. Toxicity study with SD rats was performed by WestChina-Frontier PharmaTech Co., Ltd., and the animals were kept in their facility. All procedures involving experimental animals were performed in accordance with protocols approved by the Institutional Animal Care and Use Committee (IACUC) of Peking University, or Fudan University, or WuXi AppTec, or WestChina-Frontier PharmaTech.

### ApoB knockdown in normal C57BL/6 mice

Apolipoprotein B (apo B) is encoded by the APOB gene and dominantly expressed in the liver. It is the primary apolipoprotein of chylomicrons, VLDL, IDL, and LDL particles, which is responsible for carrying fat molecules, including cholesterol, around the body to all cells within all tissues. Here, siRNA-against apoB (siApoB) was used to evaluate the delivery efficiency of RBP131. Male C57BL/6 mice were randomly divided to nine groups with eight mice per group. Then they were given 1 × PBS, naked siRNA, MC3/siRNA complex (positive control, PosCtl), and RBP131/siApoB complex, respectively. The dose of naked siRNA and MC3/siRNA were 1 and 0.1 mg/kg, respectively. The doses of RBP131/siRNA were 1.0, 0.5, 0.25, 0.1, 0.05, 0.01 mg/kg, respectively. After 72 h, animals were sacrificed by cervical isolation. Blood samples were collected by retroorbital eye bleed. Tissue samples were subjected to RNAlater^®^ (Sigma–Aldrich), kept at 4 °C for 24 h, and then transferred to −80 °C for storage. Subsequently, tissues were homogenized, followed by adding RNAVzol (Vigorous Biotechnology Beijing Co., Ltd., Beijing, China) and extracting total RNA according to the manufacturer’s protocol. After finishing reverse transcription with total RNA (1 μg), cDNA (50 ng) was quantified by RT-PCR system using SYBR Green PCR Master Mix. The expression level of apoB or HBV gene was analyzed using the Ct (cycle threshold) values with the standard protocol. β-actin was selected as the reference gene. Then a fitting sigmoid curve for apoB mRNA knockdown was generated with GraphPad Prism software according to a function of Y = Bottom + (Top-Bottom)/(1 + 10^((LogIC_50_-X) × HillSlope)), by which the ED_50_ of RBP131/siApoB was calculated.

To evaluate lipid accumulation in the liver resulting from the knockdown of the apoB gene, lipids in the liver were extracted and detected. When mice were sacrificed, a piece of the liver was collected and stored at −80 °C. Then 50 mg of the liver tissue was measured with analytical balance (Mettler–Toledo International, Inc., Columbus, OH, USA), followed by adding 1 ml solution of chloroform and methanol (2:1, v/v), homogenizing with homogenizer (Superfine Homogenizers, Fluko^®^, Essen, Germany), and keeping at 4 °C overnight. Then 0.3 ml of deionized water was added into the sample, followed by mixing with vortex and centrifuging for 10 min at 8,000 rpm at 4 °C (Eppendorf^®^ Centrifuge 5417 R, Eppendorf, Hamburger, Germany). Then solution at the organic phase (~200 μl) was collected and volatilized to dryness by centrifuging with concentrator 5301 (Eppendorf^®^, Hamburger, Germany). Subsequently, the dried sample was re-dissolved with 600 μl of 1 × PBS containing 5% (v/v) triton, incubated at 60 °C for 30 min, and ultrasonically treated for 30 min. The procedures of incubation at 60 °C and ultrasonic treatment were repeated once until the lipids were totally dissolved. Finally, the TG and CHO levels in the liver were detected with commercial detecting Kits (Biosino Bio-Technology and Science Incorporation, Beijing, China) according to the manufacturer’s protocols.

In parallel, pieces of the livers were embedded with OCT. Eight-micrometer cryosections were prepared and stained with Oil Red O. Then they were observed using an inverted fluorescence microscope (Olympus X71, Olympus, Tokyo, Japan), from which the lipid accumulation was also analyzed.

In addition, serum samples were analyzed by Beijing DIAN Clinical Laboratory Co. Ltd., which is a company providing preclinical and clinical analysis services. Concentrations of TG and CHO in serum were detected with a biochemistry analyzer.

To evaluate the longevity of gene silencing triggered by RBP131/siRNA, a duration assay was performed. Three groups of mice were given RBP131/siApoB complex at the doses of 1.0, 0.5, and 0.25 mg/kg, respectively. Normal saline was included as a control. The concentration of total cholesterol (CHO) was examined on days 2, 4, 7, 14, 21, and 28 post-administrations according to the above-mentioned methods.

### Gene knockdown with ApoE^−/−^ and LDLR^−/−^ knockout mice

In order to explore the mechanism of RBP131 in mediating siRNA delivery into hepatocytes, wild type C57BL/6, ApoE^−/−^ (ApoE knockout), and LDLR^−/−^ (LDLR knockout) mice were employed. Three kinds of mice were divided into four groups (six mice per group), respectively. For each kind of mice, RBP131/siApoB was dosed at 0.5, 0.1, and 0.05 mg/kg, respectively. 1 × PBS was included as a negative control. Expression level of apoB mRNA was analyzed at 72 h post-administration by quantitative RT-PCR.

### Inhibition of HBV mRNA expression in transgenic mice

HBV transgenic mice C57BL/6J-TgN(AlblHBV) 44Bri/J containing HBV genome S, pre-S and X domains,^46^ were from Peking University Health Science Center (Beijing, China). Seven to ten-week-old mice were employed to evaluate the gene silencing efficiency of anti-HBV siRNA (siHBV) encapsulated by RBP131. Here, six groups of mice (eight animals per group, with half males and half females) were dosed with saline, naked siHBV, MC3-loaded siHBV (MC3/siHBV), empty RBP131, RBP131-loaded scramble siRNA (or NC siRNA), and RBP131-loaded siHBV (RB-HBV008), respectively. The dose of siRNA was 1 mg/kg. The dose of empty RBP131 (lipids) was the same as the dose of lipids contained in RB131/siHBV complex. Seventy-two hours later, mice were sacrificed by cervical dislocation. Liver samples were collected and the expression level of HBV gene was analyzed according to the above-mentioned protocol.

### In vivo efficacy of RB-HBV008 in disease models

To evaluate in vivo anti-HBV efficacy of RB-HBV008, three HBV mouse models were applied. The first one is C57BL/6J-TgN(AlblHBV) 44Bri/J, which is a transgenic mouse model containing partial HBV genome, as described before. The second one is C57BL/6J-M-Tg (HBV C1.0), which is another transgenic mouse model containing 1.0 copies of HBV genome. It can generate most virus elements, e.g., HBsAg and HBcAg. However, it cannot product the HBV virion. The third one is pAAV-HBV mouse model. This model is established by hydrodynamic injection of pAAV-HBV1.3mer plasmid DNA that contains a 1.3-fold-overlength genome of HBV. It can generate all virus elements, such as HBsAg, HBeAg, HBV DNA, as well as virions. For efficacy evaluation in C57BL/6J-M-Tg (HBV C1.0) and pAAV-HBV mouse models, single or multiple doses of RBP131/siRNA formulation were intravenously injected into the mice at indicated dosages, which was shown in the figures or figure legends. For multiple dosing experiments, siRNA was weekly or biweekly injected into the mice, which was also shown in the figure legends. At indicated time points after treatment, serum and/or tissue samples were collected. Multiple parameters, e.g., HBsAg, HBeAg, virus DNA, and virus mRNA, were analyzed by ELISA or real-time PCR.

### Toxicity study in rodent

Male CD-1 mice were randomly divided into seven groups with eight mice per group. Then they were given following formulations at the dose volume of 10 mL/kg respectively: (1) without treatment; (2) 1 × PBS, (3) lipopolysaccharides (LPS) (5 mg/kg); (4) poly I:C (10 mg/kg); (5) RBP131/siRNA complex (1 mg/kg); (6) RBP131/siRNA complex (3 mg/kg); (7) RBP131/siRNA complex (5 mg/kg). LPS and poly I: C were administered via intraperitoneal (i.p.) injection. Other samples were administered *via* intravenous (i.v.) injection. Blood samples were collected at 3, 24, and 48 h after injection from the animals. Weights of the body, the liver, and the spleen were recorded to calculate organ coefficients. Immunoassays were used to measure the levels of cytokines (TNF-α, IFN-γ, IL-1β, IL-2, IL-5, IL-6, IL-12(p70), MCP-1, KC, GM-CSF) in a 96-well plate using Luminex^®^ assays (high-throughput multiplex bead-based assays) with Luminex 100 system (Luminex Corporation, Austin, Texas, USA). Clinical chemistry of ALT (alanine transaminase), AST (aspartate transaminase), CRE (creatinine), and BUN (blood urea nitrogen) was measured by biochemical analyzer. Meanwhile, livers of the mice sacrificed at 48 h were fixed in 10% formaldehyde, embedded in paraffin, sectioned with Microtome (Leica, Germany), stained with H&E, and at last observed histological change with an optical microscope (Olympus X71, Olympus, Tokyo, Japan).

Toxicity evaluation was also performed with SD rats. Animals were divided into two groups with 10 animals (5 males and 5 females) per group. One group received 1 × PBS treatment, another received three doses of RBP131-formulated siRNA at the dose of 5 mg/kg. Three doses were administrated on day 1, day 6, and day 12, and the experiment was terminated on day 14. Dosing volume was kept at 20 mL/kg. When the animals were sacrificed on day 14, blood biochemistry was analyzed. In addition, the main organs include of the liver were fixed with 10% formaldehyde, then embedded in paraffin, at last, observed histological change with an optical microscope.

### Statistical analysis

The data were expressed as the mean ± SD or mean ± SEM, as indicated in figure legends. One-way or two-way analysis of variance (ANOVA) was used when there were multiple comparisons. A two-tailed Student’s *t* test was used for single comparisons. The specific statistical methods are indicated in the figure legends. Significance was defined as *P < 0.05, **P < 0.01, and ***P < 0.001.

## Supplementary information

Supplementary Information

## Data Availability

All data that support the findings of this study are available from the corresponding author upon reasonable request.
