# Supplementary material for: Ionizable liposomal siRNA therapeutics enables potent and persistent treatment of Hepatitis B
Source: Signal Transduct Target Ther. 2022 Feb 11;7:38. doi: 10.1038/s41392-021-00859-y (PMC8831581; doi:10.1038/s41392-021-00859-y)
Supplement: Supplementary file 1 — Supplementary Information [file 41392_2021_859_MOESM1_ESM.docx]

Supplementary Materials for

**Ionizable liposomal siRNA therapeutics enables potent and persistent treatment of Hepatitis B**

Yuanyu Huang^1, 2, *^, Shuquan Zheng^1^, Zhaoxu Guo^1^, Xavier de Mollerat du Jeu^3^, Xing-Jie Liang^4^, Zhiwei Yang^1^, Hong-Yan Zhang^1^, Shan Gao^1^, Zicai Liang^1, *^

^1^ Suzhou Ribo Life Science Co. Ltd., Jiangsu 215300, China.

^2^ Advanced Research Institute of Multidisciplinary Science; School of Life Science; School of Medical Technology (Institute of Engineering Medicine); Key Laboratory of Molecular Medicine and Biotherapy; Key Laboratory of Medical Molecule Science and Pharmaceutics Engineering; Beijing Institute of Technology, Beijing 100081, China.

^3^ Thermo Fisher Scientific, Carlsbad, California, USA.

^4^ Chinese Academy of Sciences (CAS) Center for Excellence in Nanoscience and CAS Key Laboratory for Biomedical Effects of Nanomaterials and Nanosafety, National Center for Nanoscience and Technology, Beijing 100190, China.

Correspondence to: Y. Huang (yyhuang@bit.edu.cn, Tel: +86-10-68911089) or Z. Liang (liangzc@ribolia.com, Tel: +86-10-62769862)

**This PDF file includes:**

Supplemental materials and methods

Fig. S1 In vivo biodistribution of RBP131/siRNA nanoparticle.

Fig. S2 Confocal imaging of the liver cryosections.

Fig. S3 APOB silencing and phenotype responses in C57BL/6j mice

Fig. S4 Inhibition of HBV gene expression in HBV-transgenic animal model (model 1).

Fig. S5 5’-RACE PCR assay for validation of RNAi mechanism in vivo.

Fig. S6 HBsAg reduction in transgenic mouse model receiving multiple doses of RB-HBV008 (model 2).

Fig. S7 Body weights and organ coefficients of mice receiving high dose of RB-HBV008 in mice.

Supplementary Text

Supplemental materials and methods

*In vivo* biodistribution

Male C57BL/6 mice, 5-7 weeks old and weighing 18-22 g, were used to investigate the biodistribution profile of systemically-administered siRNA. The abdomen was depilated with depilatory cream one day before administration to facilitate observation. Formulations were administered to each mouse *via* tail vein injection at 1.0 mg/kg (for siRNA). The fluorescence signal of cyanine Cy5 from the whole body was recorded at given time points using an imaging system (Kodak In-Vivo Imaging System FX Pro, Carestream Health, Toronto, Canada). In this experiment, 630 nm excitation and 700 nm emission filters were selected. According to the manufacturer’s specifications, the bandpasses of the excitation and emission filters were 20 nm and 60 nm (wide-angle), i.e., the excitation and emission spectra were 620-640 nm and 670-730 nm. The other conditions were as follows: exposure time, 60.0 sec; X-binning, 2× binning; Y-binning, 2× binning; f-stop, 2.50; field of view (FOV), 180 mm; focal plane, 13.0 mm. Mice were anesthetized during imaging with a mixture of oxygen and isoflurane using a vaporizer (Matrix VIP3000 Isoflurane Vaporizer, Midmark Corporation, Ohio, USA). At the end-point, mice were sacrificed by cervical dislocation, and the major organs were isolated and examined. The above experiments were independently replicated three times. Quantitative analyses were performed using a molecular imaging software package (Carestream Health, Toronto, Canada).

Tissue cryosectioning for fluorescence observation

Tissues were placed in Omnisette tissue cassettes, embedded in OCT, and frozen on a foam floater on liquid nitrogen in a pre-chilled Dewar flask for ~1 min until the OCT turned white and opaque. Then, the specimens were cut into 10 μm sections on a cryostat. Each section was picked up on a glass slide, stained with DAPI to visualize the nucleus and with phalloidin to visualize F-actin in order to display the rough outline of the cell. Finally, cryosections were examined under a confocal microscope (LSM 700, Carl Zeiss, Oberkochen, Germany).

5’-RACE PCR

To demonstrate that the RB-HBV008 downregulates targeted gene expression via RNAi mechanism in vivo, the mRNA cleavage products were characterized using a 5’-rapid amplification of cDNA ends (5’-RACE) PCR technique. Briefly, RB-HBV008 was intravenously administered into the mice, the liver tissues were collected at 7 hours, 24 hour, 48 hours, and 72 hours post injection. The total RNA was prepared, and ligated directly to 5’-adaptor sequences using T4 RNA ligase. The the ligation products were purified by phenol extraction and ethanol precipitation, followed by reverse-transcribing using reverse transcription kit. A forward primer that is complementary to the adaptor sequence (5’-CGACTGGAGCACGAGGACACTGA-3’), and HBV gene specific reverse primer (5’-GCTGTAGGCATAAATTGGTC-3’) were used to complete a PCR assay. The PCR products were separated in an agarose gel via electrophoresis, and the target PCR products were excised from the gel and sequenced directly to confirm RACE band identities.

Fig. S1.


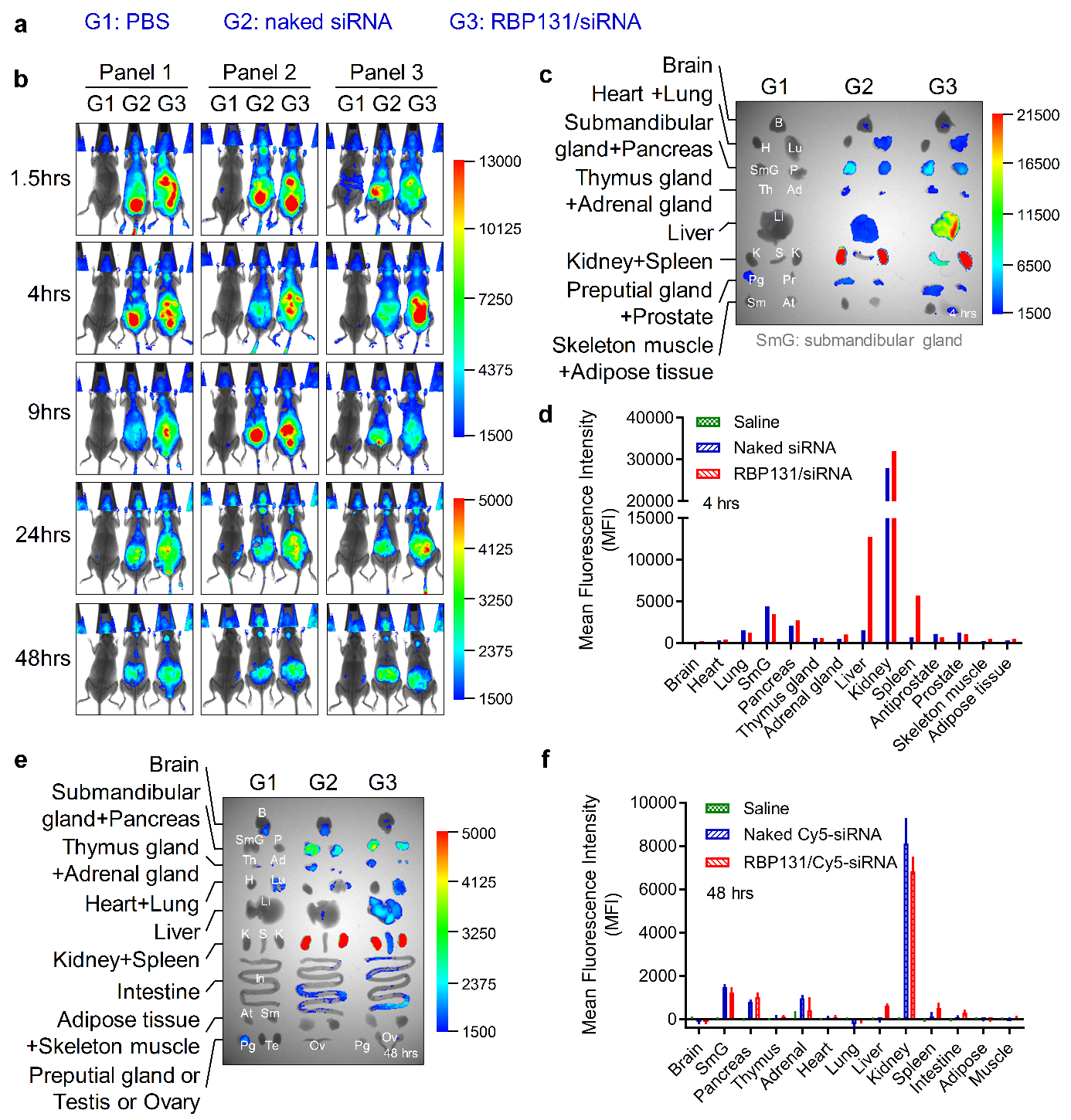


**Supplemental Fig. S1. In vivo biodistribution of RBP131/siRNA nanoparticle.** (**a**) Grouping information. Three groups of mice with three animals per group was employed in this study, which were treated with PBS, naked siRNA, and RBP131/Cy5-siRNA, respectively. (**b**) Whole-body imaging of the animals at indicated time points after administration. (**c**) Fluorescence imaging of isolated tissues at 4 hours post injection. (**d**) Quantitative analysis of (**c**). (**e**) Recording of the fluorescence signal in isolated tissues at 48 hours post injection. (**f**) Quantitative analysis of (**e**). Data were shown as the mean ± SEM. B, brain; H, heart; Lu, lung; SmG, submandibular gland; P, pancreas; Th, thymus gland; Ad, adrenal gland; Li, liver; K, kidney; S, spleen; Pg, preputial gland; Pr, prostate; Sm, skeleton muscle; At, adipose tissue; In, intestine; Te, testis; Ov, ovary.

Fig. S2.


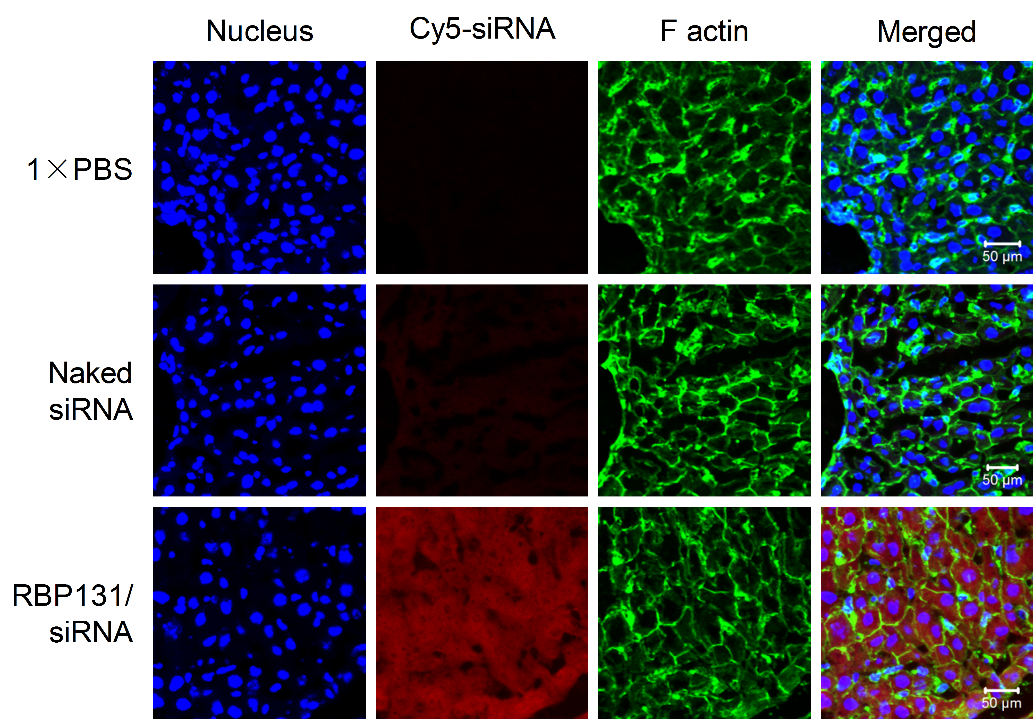


**Supplemental Fig. S2 Confocal imaging of the liver cryosections.** Mice were treated with 1×PBS, naked siRNA and RBP131-formulated siRNA, respectively. Livers were harvest at 1.5 h after intravenous injection, followed by OCT embedding, section cutting, and staining with DAPI (blue, for staining nuclei), FITC-labeled phalloidin (green, for staining F actin to show the rough cell outlines). Then these sections were observed with Confocal microscope (LSM700, Carl Zeiss). Scale bar: 50 μm.

Fig. S3.


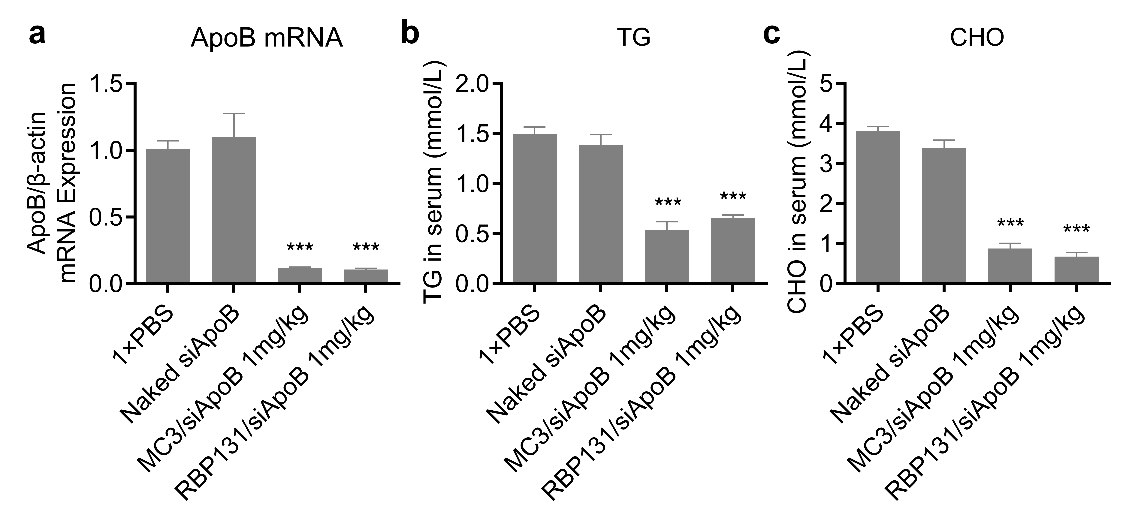


**Supplemental Fig. S3 APOB silencing and phenotype responses in C57BL/6j mice.** (**a**) mRNA expression detected by RT-qPCR. (**b** and **c**) Levels of total triglyceride (**b**) and total cholesterol (**c**) in serum. siRNA was dosed at 1 mg/kg via intravenously injection. Samples were harvested three days later.

Fig. S4.


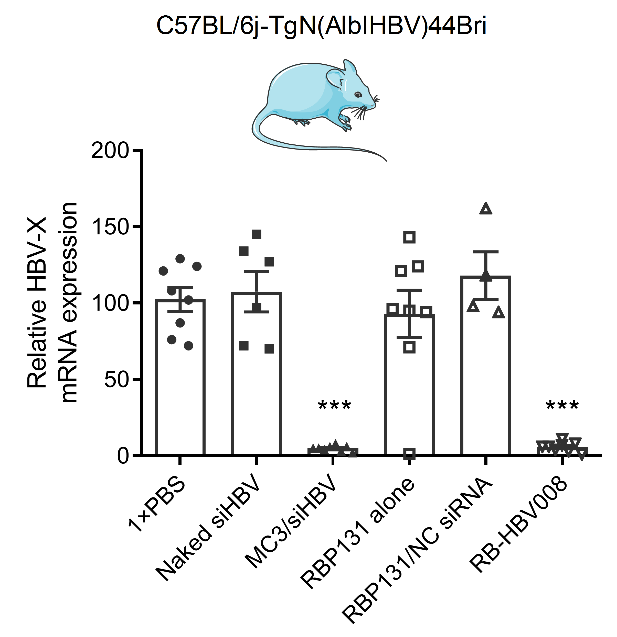


**Supplemental Fig. S4 Inhibition of HBV gene expression in HBV-transgenic animal model (model 1)**. Single dose of various formulations, including 1 × PBS, naked siHBV, MC3-loaded siHBV, empty RBP131 carrier, RBP131-loaded scramble siRNA (NC siRNA), and RB-HBV008, were intravenously administered to HBV transgenic mice (C57BL/6j-TgN(AlblHBV)44Bri). Data revealed that the expression of X gene of HBV was robustly inhibited for the mice treated with MC3/siHBV and RB-HBV008.

Fig. S5.


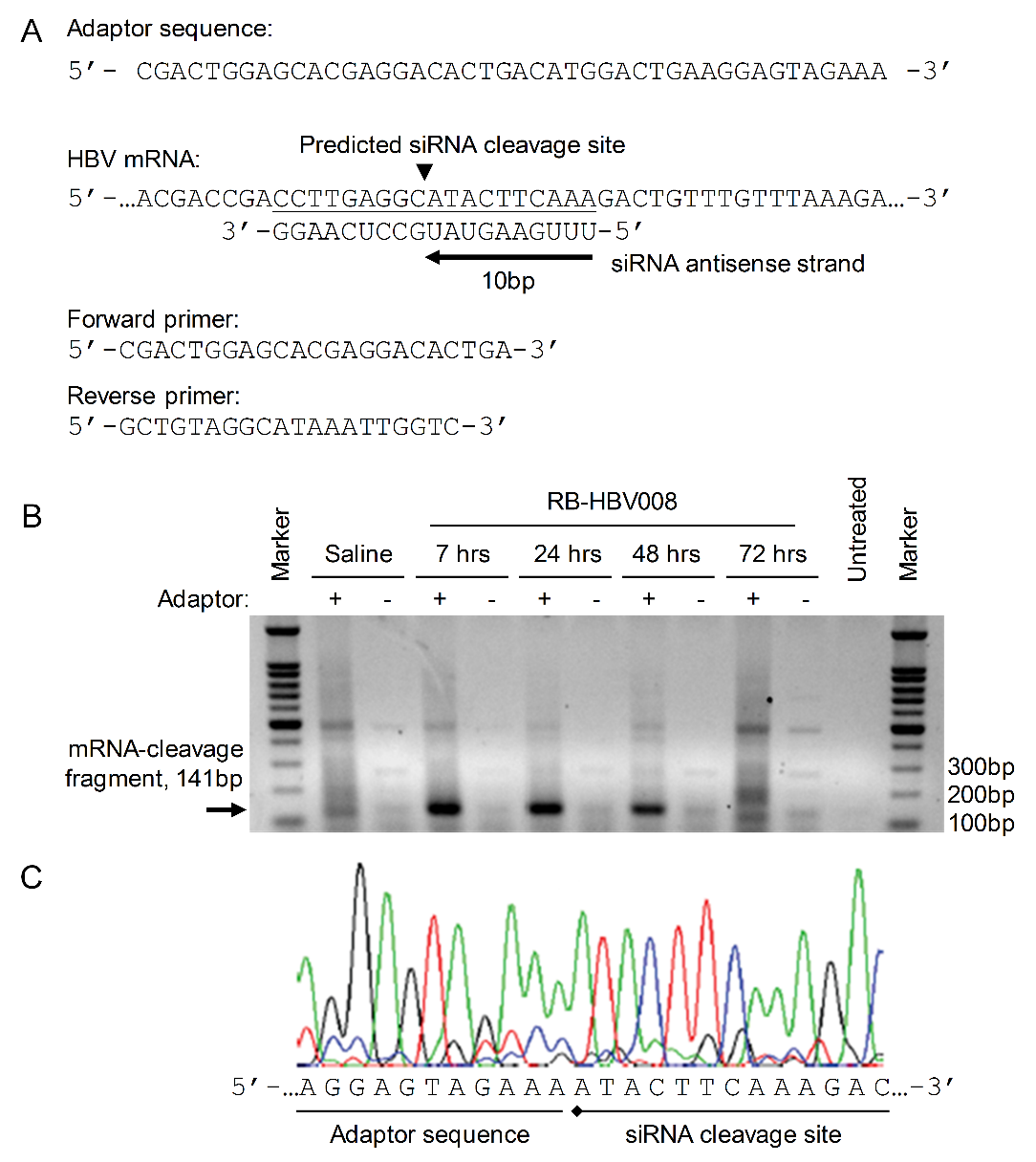


**Supplemental Fig. S5 5’-RACE PCR assay for validation of RNAi mechanism *in vivo*.** RB-HBV008 was intravenously injected to the mouse model of C57BL/6j-TgN(AlblHBV)44Bri. The animals were sacrifice at 7 hours, 24 hours, 48 hours, and 72 hours after injection, and the liver tissues were collected. Then total RNA was extracted, followed by performing 5’-RACE-PCR. The products were analyzed by electrophoresis separation.

Fig. S6.


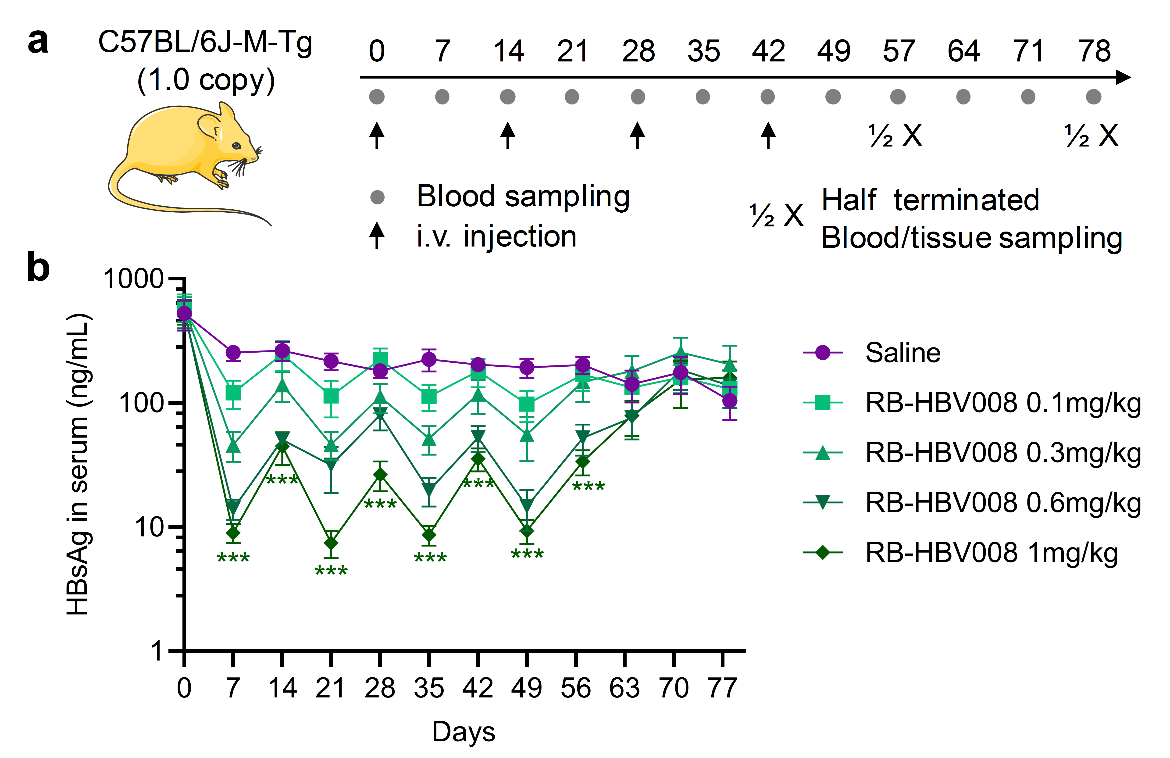


**Supplemental Fig. S6 HBsAg reduction in transgenic mouse model receiving multiple doses of RB-HBV008 (model 2).** (**a**) Treatment and sampling schedule of “1.0M-MD2” study, in which RB-HBV008 was intravenously injected into the model mice at the doses of 1.0 mg/kg, 0.6 mg/kg, 0.3 mg/kg and 0.1 mg/kg, respectively. Blood samples were collected every two weeks, and the study was terminated at day 78 after treatment. (**b**) HBsAg expression during the treatment course.

Fig. S7.


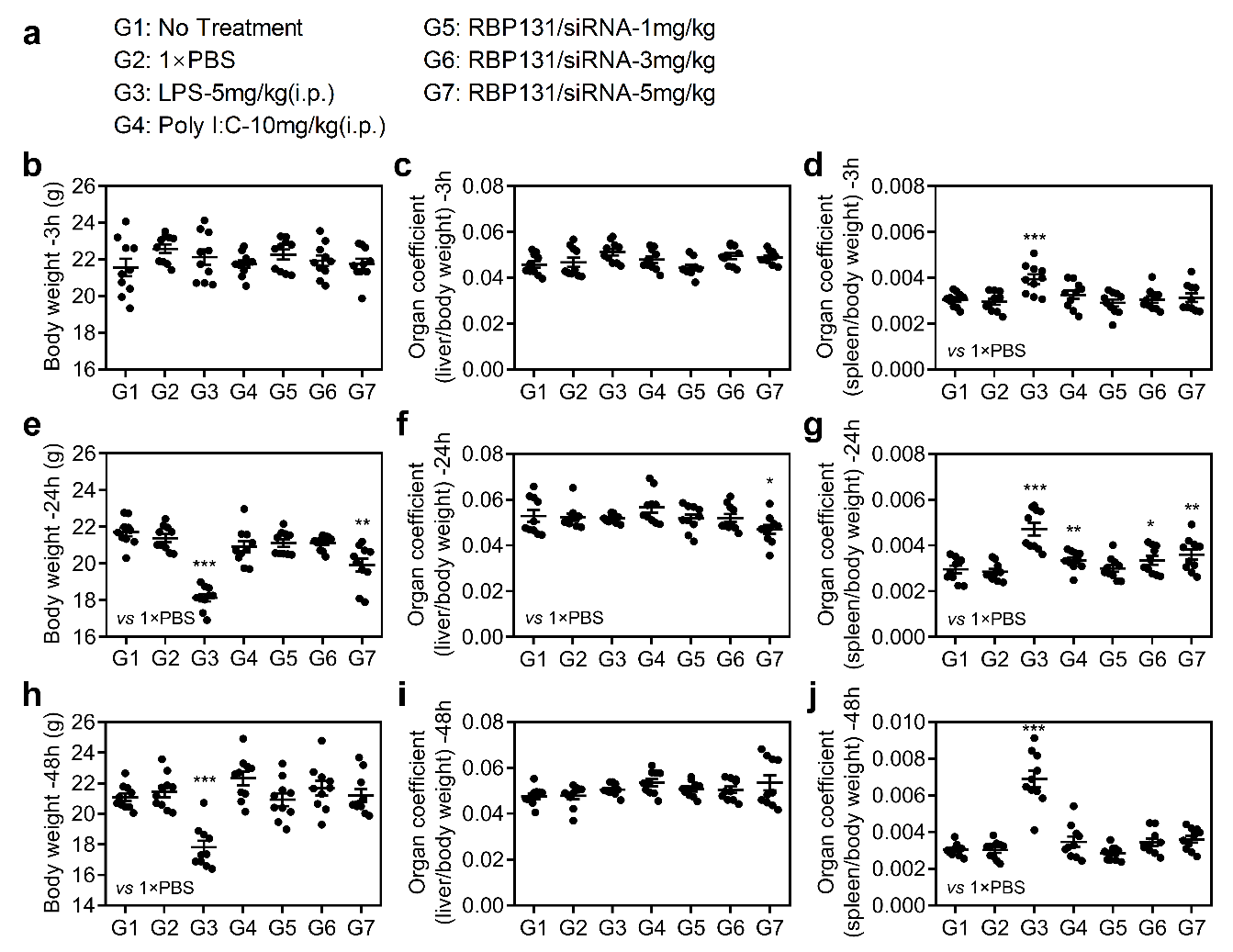


**Supplemental Fig. S7 Body weights and organ coefficients of mice receiving high dose of RB-HBV008 in mice.** (**a**) Grouping information. CD-1 mice were divided to seven groups, which received no treatment (G1), or were treated with 1 × PBS (G2), lipopolysaccharide (LPS, G3), Poly I:C (G4), or RB-HBV008 at the dosages of 1 mg/kg (G5), 3 mg/kg (G6) and 5 mg/kg (G7), respectively. (**b**-**d**) The body weight (**b**), organ coefficient of the liver (**c**) and organ coefficient of the spleen (**d**) recorded at 3 hours after injection. (**e**-**g**) The body weight (**e**), organ coefficient of the liver (**f**) and organ coefficient of the spleen (**g**) recorded at 24 hours after injection. (**h**-**j**) The body weight (**h**), organ coefficient of the liver (**i**) and organ coefficient of the spleen (**j**) recorded at 48 hours after injection.
